# Global, regional and national cancer burden and trends of middle-aged and older populations: a GBD 2021-based analysis, prediction and prevention

**DOI:** 10.3389/fonc.2026.1859604

**Published:** 2026-10-07

**Authors:** Ruokai Sun, Feng Shi, Yinsi Tang, Huicong Song, Jiamin Song, Lirong Yan, Yang Chen, Hongfei Yan

**Affiliations:** 1 Department of Medical Oncology, The First Hospital of China Medical University, Shenyang, Liaoning, China; 2 Department of Health Management, Shengjing Hospital of China Medical University, Shenyang, China; 3 Department of Gastroenterology, The Fourth Affiliated Hospital of China Medical University, Shenyang, China; 4 Department of Oncology, The Affiliated Hospital of Chifeng University, Chifeng, Inner Mongolia, China; 5 Department of Respiratory and Infectious Disease of Geriatrics, The First Hospital of China Medical University, Shenyang, China; 6 Sichuan Provincial People’s Hospital, University of Electronic Science and Technology of China, Chengdu, China; 7 Department of Medical Oncology, Chongqing University Cancer Hospital, Chongqing, China; 8 Provincial Key Laboratory of Anticancer Drugs and Biotherapy of Liaoning Province, The First Hospital of China Medical University, Shenyang, Liaoning, China; 9 Clinical Cancer Research Center of Shenyang, The First Hospital of China Medical University, Shenyang, Liaoning, China; 10 Key Laboratory of Precision Diagnosis and Treatment of Gastrointestinal Tumors (China Medical University), Ministry of Education, Shenyang, Liaoning, China

**Keywords:** cancer burden, cancer trends, global burden of diseases (GBD), middle-aged and older adults, risk factors

## Abstract

**Background:**

Middle-aged and older adults (MAOs; defined as individuals aged ≥40 years) account for the global cancer burden, contributing to 70–80% of cases. Characterizing cancer profiles in this demographic is essential for developing targeted prevention and treatment strategies to mitigate global impacts.

**Methods:**

Using Global Burden of Diseases (GBD) 2021 data, we analyzed incidence, prevalence, mortality and Disability-Adjusted Life Years (DALYs) for 31 cancers among MAOs (aged≥40) across 204 countries/21 regions(1990-2021). Trends were quantified via Joinpoint regression, and autoregressive integrated moving-average (ARIMA) models projected the burden through 2035. Risk attribution utilized population attributable fractions across Socio-demographic Index (SDI) quintiles. An order-independent Shapley decomposition attributed projected cancer incidence changes during 2021–2035 to population growth, aging, and age-specific incidence rate shifts.

**Results:**

In 2021, MAOs accounted for 48,804,114 incident cases and 9,434,210 deaths. Females exhibited a 1.14-fold higher age-standardized incidence rate (ASIR) than males, yet males had a higher age-standardized mortality rate (ASMR). Projections suggested a continued rise in cancer burden through 2035. Peak incidence occurred in ages 60–69 and the highest mortality in ages 70–79. Breast cancer was the main cancer in females; tracheal/bronchus/lung cancer in males. Tracheal/bronchus/lung cancer dominated mortality across all groups except middle-aged women (breast cancer). High-SDI regions bore the greatest burden, characterized by tracheal/bronchus/lung and colorectal cancers, while low-SDI regions shifted toward breast, cervical and stomach cancer. Risk factor analysis from 2010–2021 revealed rising metabolic risks, with high body mass index (BMI) and fasting plasma glucose-attributable DALYs increasing by 12.39% and 11.36% respectively, especially in high-SDI regions. Conversely, while most behavioral risks declined, smoking remained the leading risk factor, contributing over 60% to lung cancer DALYs. In low-SDI regions, behavioral drivers such as unsafe sex (accounting for >90% of cervical cancer risk) and air pollution remained the dominant persistent risks.

**Discussion:**

This study, the first focusing on MAOs’ global cancer burden, reveals heterogeneous patterns by age, gender, and SDI. Lung cancer was the leading cause of death (smoking as main risk). Metabolic risks rose in high-SDI regions, behavioral risks in low-SDI regions. In conclusion, MAOs’ cancer burden will rise through 2035; region-specific precision strategies are essential.

## Introduction

As of 2023, the latest statistics from the World Health Organization (WHO) and the International Agency for Research on Cancer (IARC) indicate that cancer ranks as the second leading cause of both incidence and mortality globally, surpassed only by cardiovascular diseases (1, 2). Reducing cancer incidence and achieving its eradication remain critical public health priorities worldwide. Cancer incidence varies significantly across age groups, with a marked increase in prevalence among middle-aged and older populations. Specifically, the incidence of cancer in children and adolescents under 15 years of age is relatively low, accounting for approximately 1% of total cases, with hematological malignancies and central nervous system tumors being the most common (3). Among adolescents and young adults (AYAs) aged 15–39, cancer incidence remains modest, representing about 5% of total cases, with thyroid cancer, breast cancer, hematological disorders, and testicular cancer being the predominant types, which has been fully characterized (4, 5). To distinguish this population from adolescents and young adults (AYAs), conventionally defined as individuals aged 15–39 years, we defined middle-aged and older adults (MAOs) as individuals aged ≥40 years. This pragmatic cutoff is consistent with age-stratified Global Burden of Disease (GBD) burden analyses (6), while individuals aged ≥40 years account for most cancer cases and experience increasing incidence with age (7, 8). Therefore, a comprehensive analysis of the cancer burden, distribution, trends, and risk factors among individuals aged 40 and above is essential for developing effective strategies to reduce the global cancer burden.

Cancer incidence is influenced by a multitude of environmental and socioeconomic factors, including lifestyle, healthcare accessibility and socioeconomic conditions, leading to significant geographical disparities in cancer distribution. Research highlights that High-income countries, characterized by advanced healthcare systems and robust health awareness initiatives, tend to exhibit higher cancer incidence rates but lower mortality rates. Conversely, middle- and low-income countries, often constrained by limited healthcare resources, experience higher cancer mortality rates (7, 9). Breast cancer, for instance, is the leading cause of cancer incidence and mortality among women globally. A study by the International Agency for Research on Cancer involving 2 million women across 81 countries revealed that nearly one-third of breast cancer cases in sub-Saharan Africa are diagnosed at an advanced stage, compared to only one-tenth in Europe and North America (10). Similarly, lung cancer remains one of the most prevalent and deadly cancers worldwide. In China, the annual incidence rates for men and women are approximately 50 and 22 cases per 100,000 people, respectively, while in India, the rates are significantly lower, at 6 and 2 cases per 100,000 people (11, 12). These disparities underscore the importance of understanding the cancer burden among MAOs across diverse regions to inform targeted interventions and improve survival outcomes.

The GBD database, which encompasses data from 204 countries and regions, provides a comprehensive resource for analyzing disease patterns, including cancer. Recent updates to the GBD database have enhanced its accuracy and coverage, offering more precise estimates of the global cancer burden (13). Despite these advancements, no studies have yet utilized this updated dataset to specifically assess the cancer burden in MAOs. This study aims to address this gap by evaluating the global burden and trends in cancer among individuals aged 40 and above, with a focus on incidence and mortality rates, as well as disparities across gender, geography, and levels of human development. The findings will provide critical evidence to guide the formulation of targeted cancer control strategies and the equitable allocation of healthcare resources for this substantial and vulnerable population.

## Methods

### Date sources and processing

Data on 31 cancer types (**Table 1**) were extracted from the GBD 2021 for MAOs, stratified into five age groups (40–49, 50–59, 60–69, 70–79, and ≥80 years) and by gender, across 204 countries/territories (1990-2021) (**Supplementary Tables S1**, **S2**). Notably, breast cancer data included both male and female cases, and all relevant burden analyses were performed under gender stratification. Analysis adheres to GBD’s methodological practice for capturing cancer risk accumulation patterns starting at 40 years old. Socio-demographic Index (SDI) scores and population denominators were integrated from GBD metadata and United Nations World Population Prospects 2022 (14), respectively. For the decomposition analysis, age- and gender-specific population estimates for 2021 and medium-variant population projections for 2035 were obtained from the United Nations World Population Prospects 2024. Ethical approval was granted exemption under GBD guidelines (University of Washington Institutional Review Board (#46687)), as data were aggregated and anonymized.

**Table 1:** Global cancer burden in MAOs: incidence/mortality, 1990–2021.

Location_name	Cause	Incidence					Deaths				
		Number (95% UI)		ASIR (95% UI)		EAPC (95% UI)	Number (95% UI)		ASMR (95% UI)		EAPC (95% UI)
		1990	2021	1990	2021		1990	2021	1990	2021	
Global	Bladder cancer	253316.52 (232483.48-267012.41)	530203.53 (480405.14-579535.52)	18.06 (16.57-19.04)	18.25 (16.54-19.95)	-0.09 (-0.13 to -0.04)	121428 (109392.65-129131.47)	220212.69 (196793.82-243826.86)	8.66 (7.8-9.21)	7.58 (6.78-8.39)	-0.59 (-0.67 to -0.5)
Global	Brain and central nervous system cancer	105083.46 (91371.28-117104.33)	266746.06 (229775.52-304841.32)	7.49 (6.51-8.35)	9.18 (7.91-10.49)	0.66 (0.63 to 0.68)	92167.44 (79436.73-103653.11)	213681.17 (184088.9-244520.56)	6.57 (5.66-7.39)	7.36 (6.34-8.42)	0.33 (0.3 to 0.37)
Global	Breast cancer	787523.91 (742048.49-825108.3)	1940772.84 (1764971.11-2095296.01)	56.14 (52.9-58.82)	66.82 (60.76-72.14)	0.49 (0.45 to 0.52)	328451.26 (306582.7-347482.12)	632143.99 (568421.1-683496.35)	23.42 (21.86-24.77)	21.76 (19.57-23.53)	-0.36 (-0.43 to -0.28)
Global	Cervical cancer	301788.53 (277958.94-326505.65)	519250.41 (467481-571416.33)	21.52 (19.82-23.28)	17.88 (16.09-19.67)	-0.58 (-0.69 to -0.47)	183779.52 (167319.07-201345.14)	266749.66 (239150.39-293273)	13.1 (11.93-14.35)	9.18 (8.23-10.1)	-1.19 (-1.31 to -1.07)
Global	Colon and rectum cancer	875199.6 (819333.36-916343.73)	2123942.53 (1908621.12-2312553.95)	62.4 (58.41-65.33)	73.12 (65.71-79.62)	0.44 (0.4 to 0.47)	546825.49 (507074.6-576712.65)	1017849.83 (910721.12-1105590.47)	38.98 (36.15-41.12)	35.04 (31.35-38.06)	-0.44 (-0.51 to -0.38)
Global	Esophageal cancer	345371.74 (304368.62-382756.66)	568365.36 (493978.99-647712.86)	24.62 (21.7-27.29)	19.57 (17.01-22.3)	-0.97 (-1.08 to -0.86)	348192.21 (306561.02-385820.93)	532331.45 (462595.35-605772.31)	24.82 (21.86-27.51)	18.33 (15.93-20.86)	-1.24 (-1.36 to -1.11)
Global	Eye cancer	10180.32 (8109.99-12775.73)	22423.34 (17310.65-27757.01)	0.73 (0.58-0.91)	0.77 (0.6-0.96)	0.24 (0.17 to 0.32)	3964.45 (3246.43-4656.62)	7077.25 (5446.34-8759.62)	0.28 (0.23-0.33)	0.24 (0.19-0.3)	-0.52 (-0.62 to -0.42)
Global	Gallbladder and biliary tract cancer	105382.94 (93437.82-115996.36)	213213.63 (173896.8-242992.09)	7.51 (6.66-8.27)	7.34 (5.99-8.37)	-0.15 (-0.25 to -0.06)	96819.75 (85161.87-107819.98)	169801.07 (138129.18-194494.77)	6.9 (6.07-7.69)	5.85 (4.76-6.7)	-0.65 (-0.74 to -0.55)
Global	Hodgkin lymphoma	24095.7 (19646.89-26795.91)	33521.2 (28006.56-39486.09)	1.72 (1.4-1.91)	1.15 (0.96-1.36)	-1.33 (-1.36 to -1.3)	16547.99 (12377.46-19032.62)	17804.85 (13802.52-22627.76)	1.18 (0.88-1.36)	0.61 (0.48-0.78)	-2.26 (-2.37 to -2.14)
Global	Kidney cancer	140301.82 (134259.85-145554.75)	360630.27 (333699.32-381518.4)	10 (9.57-10.38)	12.42 (11.49-13.13)	0.69 (0.6 to 0.79)	70865.76 (67488.57-73678.52)	154870.59 (141646.13-164519.33)	5.05 (4.81-5.25)	5.33 (4.88-5.66)	0.13 (0.1 to 0.16)
Global	Larynx cancer	122261.91 (115093.18-129420.21)	197468.55 (181898.56-214138.58)	8.72 (8.21-9.23)	6.8 (6.26-7.37)	-0.97 (-1.1 to -0.85)	83919.41 (78053.76-89823.82)	115422.08 (105984.29-125533.96)	5.98 (5.56-6.4)	3.97 (3.65-4.32)	-1.52 (-1.65 to -1.38)
Global	Leukemia	178670.38 (162103.38-191446.36)	342982.2 (293394.5-378245.11)	12.74 (11.56-13.65)	11.81 (10.1-13.02)	-0.33 (-0.39 to -0.28)	141142.02 (126208-153650.13)	248652.76 (212419.74-275457.24)	10.06 (9-10.95)	8.56 (7.31-9.48)	-0.57 (-0.61 to -0.54)
Global	Lip and oral cavity cancer	159981.53 (151479.29-168349.87)	392354.24 (356581.78-422450.68)	11.41 (10.8-12)	13.51 (12.28-14.54)	0.49 (0.39 to 0.6)	91685.77 (86018.06-97517.07)	198564.59 (178011.69-215390.03)	6.54 (6.13-6.95)	6.84 (6.13-7.42)	0.05 (-0.05 to 0.15)
Global	Liver cancer	217297.42 (196507.2-240801.77)	500484.69 (442443.83-564847.06)	15.49 (14.01-17.17)	17.23 (15.23-19.45)	0.25 (0.16 to 0.33)	215865.38 (194756.09-239844.43)	461817.1 (408642.98-519929.33)	15.39 (13.88-17.1)	15.9 (14.07-17.9)	0.01 (-0.08 to 0.1)
Global	Malignant neoplasm of bone and articular cartilage	22526.09 (20495.66-26164.18)	56020.54 (43047.95-65778.6)	1.61 (1.46-1.87)	1.93 (1.48-2.26)	0.77 (0.67 to 0.87)	21859.67 (19795.05-25660.67)	48113.2 (37437.59-55695.61)	1.56 (1.41-1.83)	1.66 (1.29-1.92)	0.31 (0.22 to 0.41)
Global	Malignant skin melanoma	99795.06 (94328-104033.03)	269754.95 (243931.33-287911.49)	7.11 (6.72-7.42)	9.29 (8.4-9.91)	0.9 (0.61 to 1.2)	29269.74 (26745.09-31011.89)	57650.16 (49877.81-62940.98)	2.09 (1.91-2.21)	1.98 (1.72-2.17)	-0.15 (-0.24 to -0.05)
Global	Mesothelioma	15795.36 (14482.67-17196.48)	30816.48 (27912.28-33163.11)	1.13 (1.03-1.23)	1.06 (0.96-1.14)	-0.02 (-0.11 to 0.07)	14500.87 (13297.46-15768.79)	28725.59 (25952.22-30977.74)	1.03 (0.95-1.12)	0.99 (0.89-1.07)	0.05 (-0.04 to 0.14)
Global	Multiple myeloma	54696.79 (50319.53-59234.3)	146218.04 (126091.14-162921.37)	3.9 (3.59-4.22)	5.03 (4.34-5.61)	0.74 (0.65 to 0.82)	46841.96 (42876.96-50977.53)	114780.5 (98163.83-128949.18)	3.34 (3.06-3.63)	3.95 (3.38-4.44)	0.4 (0.31 to 0.49)
Global	Nasopharynx cancer	59908.34 (53208.03-66795.11)	96704.31 (83160.57-112629.75)	4.27 (3.79-4.76)	3.33 (2.86-3.88)	-1.1 (-1.32 to -0.87)	54737.32 (48534.39-60969.89)	68136.01 (59988.26-77023.01)	3.9 (3.46-4.35)	2.35 (2.07-2.65)	-2.03 (-2.25 to -1.8)
Global	Neoplasms	20666313.88 (16166266.38-26949794.38)	48804113.5 (39161974.57-62039284.25)	1473.35 (1152.53-1921.32)	1680.2 (1348.24-2135.85)	0.4 (0.34 to 0.46)	5304774.37 (4992115.39-5547174.16)	9434209.7 (8572090.38-10159486.2)	378.19 (355.9-395.47)	324.8 (295.11-349.76)	-0.59 (-0.65 to -0.54)
Global	Neuroblastoma and other peripheral nervous cell tumors	1013.76 (792.1-1280.74)	3925.84 (3116.46-4777.51)	0.07 (0.06-0.09)	0.14 (0.11-0.16)	1.83 (1.68 to 1.99)	725.99 (608.94-851.99)	2545.23 (2137.44-2841.99)	0.05 (0.04-0.06)	0.09 (0.07-0.1)	1.53 (1.41 to 1.66)
Global	Non-Hodgkin lymphoma	195859.33 (183139.62-209264.79)	519869.71 (464534.54-568928.72)	13.96 (13.06-14.92)	17.9 (15.99-19.59)	0.55 (0.42 to 0.68)	116285.29 (107959.61-126854.48)	236800.11 (211870.17-258718.45)	8.29 (7.7-9.04)	8.15 (7.29-8.91)	-0.28 (-0.38 to -0.19)
Global	Other malignant neoplasms	156045.13 (139535.47-169659.74)	365854.19 (323650.89-403713.53)	11.12 (9.95-12.1)	12.6 (11.14-13.9)	0.37 (0.16 to 0.58)	112695.55 (99291.95-123944.07)	197760.05 (173435.96-217109.76)	8.03 (7.08-8.84)	6.81 (5.97-7.47)	-0.58 (-0.8 to -0.37)
Global	Other neoplasms	11626992.05 (7233783.3-17816398.53)	26677228.15 (17338678.37-39558176.33)	828.92 (515.71-1270.17)	918.43 (596.93-1361.88)	0.27 (0.15 to 0.39)	17761.64 (14844.21-22574.69)	53936.93 (44680.47-64606.91)	1.27 (1.06-1.61)	1.86 (1.54-2.22)	1.33 (1.28 to 1.38)
Global	Other pharynx cancer	62250.65 (58040.05-66831.22)	165319.02 (153058-177345.83)	4.44 (4.14-4.76)	5.69 (5.27-6.11)	0.76 (0.65 to 0.87)	42913.68 (39281.59-46960.94)	96028.17 (87421.16-104688.08)	3.06 (2.8-3.35)	3.31 (3.01-3.6)	0.16 (0.03 to 0.29)
Global	Ovarian cancer	135043.4 (122948.04-147212.71)	258115.73 (229000.42-282870.27)	9.63 (8.77-10.5)	8.89 (7.88-9.74)	-0.41 (-0.46 to -0.36)	95030.89 (86387.91-103528.62)	177318.4 (156871.23-194441.53)	6.77 (6.16-7.38)	6.1 (5.4-6.69)	-0.48 (-0.54 to -0.43)
Global	Pancreatic cancer	202276.94 (189561.31-212932.47)	500805.71 (447005.33-543569.35)	14.42 (13.51-15.18)	17.24 (15.39-18.71)	0.61 (0.54 to 0.67)	206698.85 (193466.18-217838.61)	499324.19 (446168.58-542120.4)	14.74 (13.79-15.53)	17.19 (15.36-18.66)	0.51 (0.44 to 0.58)
Global	Prostate cancer	505157.07 (471904.64-530213.6)	1321834.98 (1192905.37-1416572.01)	36.01 (33.64-37.8)	45.51 (41.07-48.77)	0.53 (0.42 to 0.64)	211536.14 (189983.98-225719.34)	431918.43 (369727.42-471621.95)	15.08 (13.54-16.09)	14.87 (12.73-16.24)	-0.26 (-0.34 to -0.17)
Global	Soft tissue and other extraosseous sarcomas	32182.26 (27415.32-36114.55)	73000.91 (62368.49-86085.71)	2.29 (1.95-2.57)	2.51 (2.15-2.96)	0.14 (0.04 to 0.23)	21756.21 (18044.06-25032.68)	41299.7 (35050.84-49641.06)	1.55 (1.29-1.78)	1.42 (1.21-1.71)	-0.47 (-0.58 to -0.36)
Global	Stomach cancer	927260.69 (829800.81-1018679.72)	1188193.62 (1005960.65-1371139.35)	66.11 (59.16-72.62)	40.91 (34.63-47.2)	-1.63 (-1.68 to -1.57)	816032.36 (726799.88-903988.92)	930101.69 (790465.37-1066394.65)	58.18 (51.82-64.45)	32.02 (27.21-36.71)	-2.05 (-2.12 to -1.97)
Global	Testicular cancer	10334.97 (9762.41-10918.43)	29911.35 (27802.75-32278.85)	0.74 (0.7-0.78)	1.03 (0.96-1.11)	1.05 (0.94 to 1.16)	3218.66 (2999.58-3445.41)	5689.72 (5226.19-6174.88)	0.23 (0.21-0.25)	0.2 (0.18-0.21)	-0.71 (-0.87 to -0.54)
Global	Thyroid cancer	69150.33 (64759.37-74741.2)	199276.6 (177539.35-219275.62)	4.93 (4.62-5.33)	6.86 (6.11-7.55)	1.2 (1.1 to 1.31)	19982.73 (18314.85-22166.06)	41995.01 (36333.41-46235.78)	1.42 (1.31-1.58)	1.45 (1.25-1.59)	0.02 (-0.05 to 0.09)
Global	Tracheal, bronchus, and lung cancer	1105272.68 (1043212.99-1161586.52)	2252684.09 (1996819.99-2491306)	78.8 (74.37-82.81)	77.55 (68.75-85.77)	-0.08 (-0.1 to -0.06)	1056818.53 (994778.87-1113622.46)	1993925.38 (1766354.19-2203348.17)	75.34 (70.92-79.39)	68.65 (60.81-75.86)	-0.36 (-0.39 to -0.33)
Global	Uterine cancer	182188.72 (165471.52-194660.3)	458022.9 (407745.43-501843.09)	12.99 (11.8-13.88)	15.77 (14.04-17.28)	0.66 (0.57 to 0.75)	52926.49 (46384.5-57800.48)	95748.25 (83018.7-107316.98)	3.77 (3.31-4.12)	3.3 (2.86-3.69)	-0.57 (-0.7 to -0.44)

ASIR, Age-Standardized Incidence Rate; ASMR, Age-Standardized Mortality Rate; EAPC, Estimated Annual Percentage Change; 95% UI, 95% Uncertainty Interval. This table presents the global cancer incidence and mortality burden in middle-aged and older adults (MAOs) from 1990 to 2021. Incidence data include the number of cases (with 95% uncertainty intervals) and age-standardized incidence rates (with 95% uncertainty intervals), along with the estimated annual percentage change in incidence rates. Mortality data include the number of deaths (with 95% uncertainty intervals) and age-standardized mortality rates (with 95% uncertainty intervals), along with the estimated annual percentage change in mortality rates. All numerical values are presented without comma separators, and no color or shading is used in the table.

### Case and exposure definitions

The study analyzed population-level burden estimates for 31 cancer types (full list in **Table 1**), excluding non-melanoma skin cancer (C44) and benign/ill-defined neoplasms. Notably, all 31 included cancer types exclusively cover malignant neoplasms in accordance with GBD 2021 International Classification of Diseases, 10th Revision (ICD-10) classification criteria (e.g., bladder cancer corresponds to codes C67–C67.9). *In situ* and benign neoplasms of the bladder are excluded. Cancer cases were defined by histopathological or clinical confirmation based on GBD 2021 protocols. Risk factors were aligned with GBD’s Level 2 risk categories (behavioral, metabolic, environmental), including high sodium intake (>3g/day), smoking, elevated body mass index (BMI), ambient fine particulate matter (PM2.5) pollution, and occupational carcinogens, including arsenic, asbestos, beryllium, cadmium, chromium, diesel engine exhaust, nickel, polycyclic aromatic hydrocarbons, silica, benzene, formaldehyde, sulfuric acid and trichloroethylene. Asbestos is only one typical representative among these occupational risk factors. Theoretical minimum risk exposure levels (TMRELs) followed GBD-defined thresholds (e.g., sodium ≤1.5g/day, PM2.5 2.4–5.9 μg/m³). Population attributable fractions (PAFs) were calculated using GBD-derived relative risks and exposure distributions, stratified by SDI quintiles.

### Burden estimation methods of cancer burden metrics

Cancer burden metrics—including incidence, mortality, prevalence and disability-adjusted life years (DALYs)—were estimated using the Global Burden of Diseases 2021 analytical framework (Version 6.812).Disability-adjusted life years were calculated as the sum of years of life lost (YLLs) due to premature mortality and years lived with disability (YLDs). Age-standardized rates (ASRs), including the age-standardized incidence rate (ASIR), age-standardized prevalence rate (ASPR), age-standardized mortality rate (ASMR), and age-standardized DALY rate (ASDR), were computed through direct standardization to the GBD World Standard Population Structure (GBD-WSPS v3.1), enabling comparable temporal (1990-2021) and spatial (204 countries/territories) assessments. Incidence data synthesis integrated population-based cancer registries (PBCRs, 43% global coverage), verbal autopsy records, and mortality-to-incidence ratios modeled via Cause of Death Ensemble modeling (CODEm v6.1) with sociodemographic covariates. Prevalence estimates incorporated stage-stratified 10-year survival probabilities calibrated by Healthcare Access and Quality (HAQ) Index quintiles, while calculations of YLDs applied GBD disability weights (0–1 scale) validated through multinational population surveys. Uncertainty quantification combined 1,000 bootstrap iterations for sampling error with Bayesian meta-regression for between-country variance, propagated across 28 CODEm submodels through ensemble averaging. All analyses were stratified by 5-year age intervals from 40–44 years to ≥80 years, gender, GBD super-regions, SDI quintiles (Q1: <0.45; Q5: ≥0.80), and urban-rural classification per WorldPop 2020 spatial delineations.

The summary “all neoplasms” estimates were generated independently at the top level of the GBD cause hierarchy and are therefore not expected to equal the arithmetic sum of the site-specific estimates because of hierarchical modeling, population weighting, and uncertainty reconciliation.

### Sex-specific ASIR comparison

Sex-Specific all-neoplasm ASIRs in 2021 were additionally summarized across the five SDI quintiles and the 21 GBD regions. Female-to-male ASIR ratios were calculated by dividing the female ASIR point estimate by the corresponding male ASIR point estimate within each stratum. Because the publicly downloadable GBD Results data used in this study did not include the paired draw-level estimates required to propagate uncertainty for a ratio, ratio-specific 95% uncertainty intervals (UIs) were not calculated. The ratios are therefore reported as point estimates, whereas male and female ASIRs are presented with their respective 95% UIs in **Supplementary Tables S3** and **S4**. Ratios close to 1.0 were interpreted cautiously.

### Risk attribution framework

This study utilized precomputed risk-attributable DALYs from the GBD 2021 study, which were derived using PAFs calculated through the GBD Comparative Risk Assessment framework. PAFs for each risk factor-cancer pair incorporated relative risks from meta-analyses, exposure distributions modeled via Ensemble Distribution Modeling (EDM v3.2), and TMRELs with prioritizing GBD-defined thresholds where disease-specific WHO standards were unavailable (e.g., sodium ≤1.5g/day [GBD] vs. WHO general ≤2.0g/day). Attributable DALYs were aggregated by age (40–59, 60+ years old), and by SDI to assess disparities. Uncertainty intervals (95% UIs) for attributable DALYs, reflecting variance in PAFs and total DALYs, were retained from GBD’s bootstrap and Bayesian meta-regression. Analyses focused on interpreting trends in risk-specific burden rather than recalculating PAFs, with methodological details for GBD’s PAF framework GBD 2021 Risk Factors Collaborators’ protocol.

### Application of joinpoint regression

Temporal trends in age-standardized incidence, prevalence, mortality and DALY rates for all cancers combined (1990–2021) were quantified with joinpoint regression (Joinpoint v 4.9.1.0, National Cancer Institute, USA). Analyses were conducted globally, separately for males and females, across the five SDI quintiles, and across the 21 GBD regions. A log-linear model ln(rate) = α+β×calendar-year was fitted after confirming Gaussian residuals (Kolmogorov–Smirnov P > 0.05). Grid-search plus Monte Carlo permutation (4,500 permutations) detected optimal joinpoints (range 0–5 in this analysis); annual percentage changes (APCs) for each segment and the corresponding weighted average annual percentage changes (AAPCs) across the entire 1990–2021 window were computed with 95% confidence intervals (CIs). An APC/AAPC whose 95% CI excluded zero was considered statistically significant (two-sided P < 0.05). Complete AAPCs, 95% CIs, P values, joinpoint counts and calendar years, and segment-specific APCs are presented in **Supplementary Tables S5A, B**.

### ARIMA models for cancer burden projections

Burden projections for 2022–2035 were generated separately for males, females, and both sexes using autoregressive integrated moving-average (ARIMA) models fitted to annual observations from 1990 to 2021. Autocorrelation function (ACF) and partial-autocorrelation function (PACF) plots guided initial orders; the auto.arima() function (R “forecast” v 8.23.0) selected the final ARIMA (p, d, q) specification that minimized the Akaike information criterion (AIC), with Bayesian information criterion (BIC) and root-mean-square error (RMSE) as parsimony checks. Residual diagnostics comprised the Ljung-Box white-noise test (P > 0.05) and quantile–quantile normality plots; forecast uncertainty was expressed as 95% prediction intervals. All analyses were performed in R (v 4.3.2) (15); P < 0.05 indicated statistical significance. The selected model orders, diagnostic statistics, and 2035 projections are presented in **Supplementary Table S6A**.

### Decomposition of projected incident cancer cases

An order-independent Shapley decomposition was used to attribute the projected change in incident cancer cases from 2021 to 2035 to population growth, population aging, and changes in age-specific incidence rates. Sex- and age-specific incidence rates for all neoplasms across 12 age groups (40–44 to ≥95 years) were obtained from GBD 2021 for 1990–2021 and projected to 2035 using non-seasonal ARIMA models selected primarily according to the corrected Akaike information criterion (AICc), with residual autocorrelation assessed using the Ljung–Box test. Population estimates for 2021 and medium-variant projections for 2035 were obtained from the United Nations World Population Prospects 2024, with the 95–99- and ≥100-year groups combined to match the GBD ≥95-year category. Incident cases were calculated by summing the products of age-specific population counts and corresponding incidence rates, and the contribution of each component was estimated by averaging its marginal effect across all possible replacement orders. Analyses were conducted globally and across five SDI quintiles, and both-sex estimates were obtained by summing sex-specific results. SDI-specific populations were approximated using fixed 2021 national SDI classifications, and component-specific uncertainty intervals were not estimated because paired draw-level data were unavailable.

### PAF-based counterfactual exposure-reduction scenario

The causal chains between cancers and their corresponding risk factors were analyzed using the GBD 2021 framework. GBD 2021 population attributable fraction (PAF) estimates were obtained from the Comparative Risk Assessment framework. The analysis focused on the impact of reducing exposure to specific risk factors by 30% among individuals aged 40 years and older. The potential reductions in age-standardized incidence rates (ASIRs) were estimated for six cancers: tracheal, bronchus, and lung cancer; breast cancer; colon and rectum cancer; esophageal cancer; pancreatic cancer; and stomach cancer. The analysis was stratified by risk factors, including ambient particulate matter pollution, occupational exposure to asbestos, smoking, diet high in red meat, high body-mass index (BMI), high fasting plasma glucose, diet low in milk, diet low in whole grains, and high alcohol use. This 30% reduction represented a standardized counterfactual scenario relative to 2021 exposure levels rather than a predicted intervention effect or an individual-level treatment effect.

## Results

### Global MAOs cancer overview

In 2021, a total of 48,804,114 new cancer cases and 9,434,210 cancer-related deaths in MAOs were reported globally, as detailed in **Table 1**. The ASRs stood at 1,680.2 per 100,000 population for incidence and 324.8 per 100,000 population for mortality, respectively (**Table 1**). Among MAOs, the top 10 tumors with the highest incidence rates were tracheal, bronchus, and lung cancer; colon and rectum cancer; breast cancer; prostate cancer; stomach cancer; esophageal cancer; bladder cancer; non-Hodgkin lymphoma; cervical cancer and pancreatic cancer (**Figure 1A**). The primary causes of cancer-related mortality in this population were tracheal, bronchus, and lung cancer; colon and rectum cancer and stomach cancer. Further, the cancer burden exhibited distinct patterns between males and females. Sex-stratified analysis showed that the global ASIR was 1,785.92 per 100,000 population (95% UI, 1,374.39–2,354.24) in females and 1,568.36 per 100,000 population (95% UI, 1,314.36–1,917.32) in males, corresponding to a female-to-male ASIR ratio of 1.139. Detailed sex-specific ASIRs stratified by SDI quintile and GBD region are provided in **Supplementary Tables S3** and **S4**, respectively. Conversely, the ASMR was higher in males, amounting to 381.0 per 100,000 population, compared with 271.7 per 100,000 population in females (**Supplementary Table S7**). Upon further stratification of females and males among MAOs into 10-year age strata, we observed that cancer incidence burden in females exceeded that in males within the 40–49 and 50–59 years groups, whereas males exhibited higher incidence rates in all remaining age strata (**Figure 1B**). Regarding prevalence rate, females was higher before 69 years old, which was similar to incidence rate (**Supplementary Figure S1** left). In contrast, cancer mortality and DALYs rate were consistently higher in males compared to females across all age groups (**Figure 1C**; **Supplementary Figure S1** right). In females, the top 10 cancers in terms of incidence rate in 2021 were breast cancer**;** colon and rectum cancer**;** tracheal, bronchus, and lung cancer**;** cervical cancer**;** stomach cancer**;** ovarian cancer**;** pancreatic cancer**;** non-Hodgkin lymphoma**;** liver cancer and leukemia (**Supplementary Figure S1B** left). Among males, the top 10 cancers with the highest incidence rate were tracheal, bronchus, and lung cancer**;** prostate cancer**;** colon and rectum cancer**;** stomach cancer**;** esophageal cancer**;** bladder cancer**;** liver cancer**;** non-Hodgkin lymphoma**;** pancreatic cancer and leukemia (**Supplementary Figure S1B** right). However, in terms of mortality, tracheal, bronchus and lung cancer had the highest rate in both males and females.

**Figure 1:**
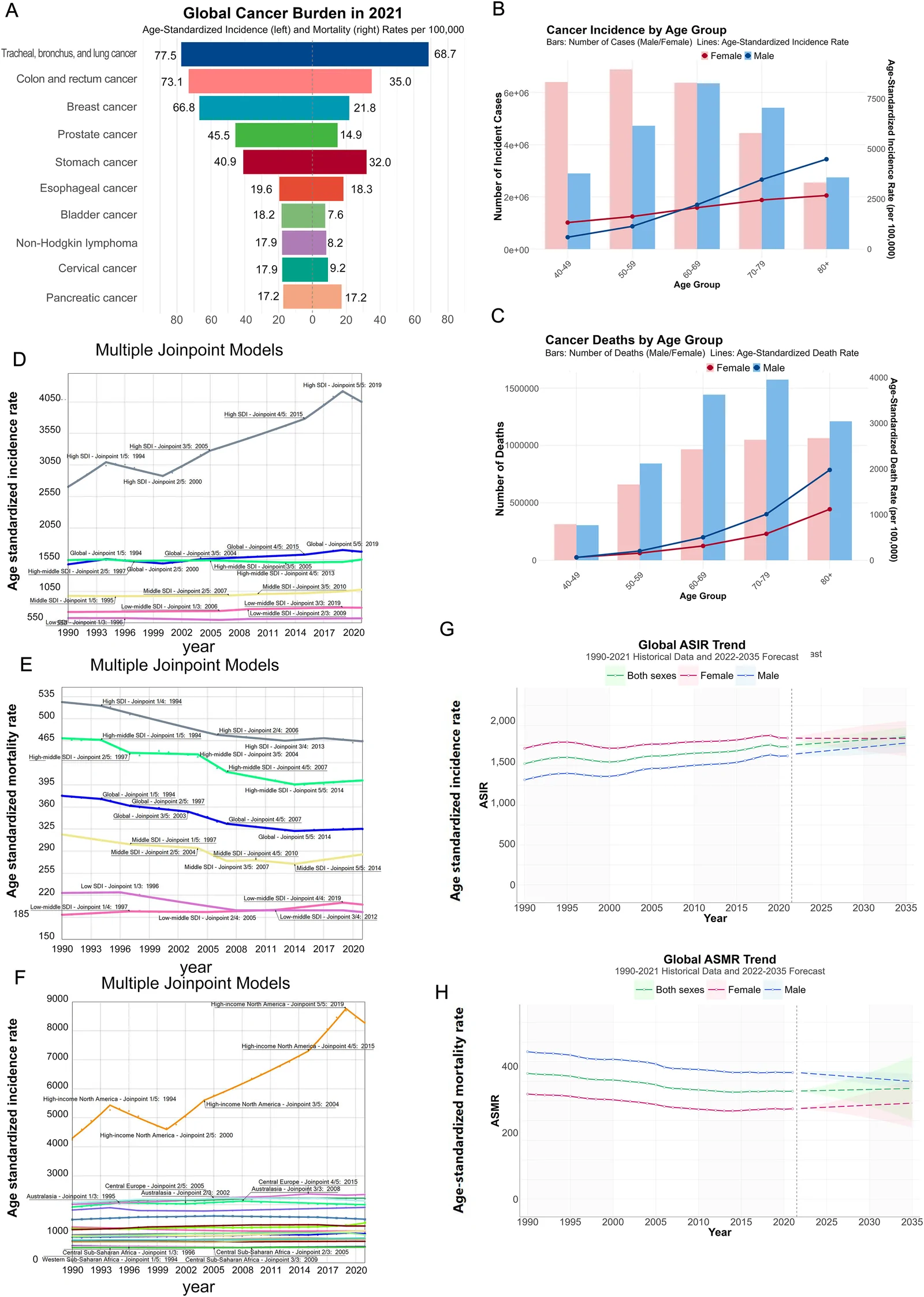
Global cancer burden of middle-aged and older adults. **(A)** Global burden of common cancers in middle-aged and older adults. **(B)** Age- stratified cancer cases and age-standardized incidence rates by gender. **(C)** Age-stratified cancer death cases and age-standardized mortality rates by gender. **(D)** Join point analysis of cancer incidence (1990-2021) by SDI. **(E)** Join point analysis of cancer mortality (1990-2021) by SDI. **(F)** Join point analysis of cancer incidence (1990-2021) by regions. **(G)** ARIMA projection of incidence during 2022-2035. **(H)** ARIMA projection of mortality during 2022-2035.

From 1990 to 2021, global ASIR and ASPR increased significantly, with AAPCs of 0.41% (95% CI, 0.35%–0.47%) and 0.48% (95% CI, 0.44%–0.53%), particularly in high-SDI regions. However, ASIR declined during 2019–2021 (APC, −0.82%; 95% CI, −1.33% to −0.31%), whereas ASPR remained stable. In contrast, ASMR and ASDR decreased overall, with AAPCs of −0.48% (95% CI, −0.55% to −0.41%) and −0.77% (95% CI, −0.83% to −0.70%), respectively, although ASMR increased slightly during the most recent period (**Figures 1D, E**, **Supplementary Figure S1C**).

ASIR and ASPR increased more rapidly in males than in females, whereas the decline in ASDR was greater in males. Recent trends differed by sex: female ASIR declined, while male ASIR remained stable; female ASMR and ASDR increased slightly, whereas the corresponding male rates remained stable or declined (**Supplementary Figures S2**, **S3**). Regionally, High-income North America had the largest increase in ASIR, whereas Central Asia had the greatest decline (**Figure 1F**).

ARIMA models showed no statistically detectable residual autocorrelation and projected the global cancer burden through 2035. ASIR was projected to reach 1,724.37 per 100,000 in males, 1,780.50 in females, and 1,798.61 for both sexes combined (**Figure 1G**), while the corresponding ASMRs were projected to be 354.25, 288.80, and 303.00 per 100,000, respectively (**Figure 1H**). ASPR was projected to increase in both sexes, whereas projected mortality and DALY trends differed by sex (**Supplementary Figure S1D**).

Incident cancer cases among adults aged ≥40 years were projected to increase from 48.56 million in 2021 to 67.80 million in 2035, representing an increase of 19.24 million cases (39.6%). Population growth accounted for 74.8% of the increase, population aging for 17.1%, and changes in age-specific incidence rates for 8.1% (**Supplementary Figure S4A**). Population growth was the predominant driver across most SDI groups, whereas incidence-rate changes contributed more substantially in high-SDI settings. The projected increase was greater in males than in females (46.4% vs 34.1%), although population growth accounted for a larger proportion of the increase in females (84.4% vs 66.1%; **Supplementary Figure S4B**; **Supplementary Table S8**).

### Differential cancer burden across age groups of MAOs

To conduct an in-depth analysis of cancer burden across different age groups, we categorized the MAOs into 5 groups as follow, 40–49 years, 50–59 years, 60–69 years, 70–79 years, and 80 years old above. Significant age-related differences in global cancer burden of MAOs were observed. Significant age-related heterogeneity in global cancer burden was observed among MAOs. The number of new cases increased steadily with age before 70 years old and gradually decreased thereafter, with the most pronounced decline in the ≥80 years group (**Figure 2A**, left). Cancer-related deaths peaked in the 70–79 years age group (**Figure 2A**, right). Upon the proportional distribution of cancer types across age groups, breast and cervical cancers constituted the predominant cancer burden in middle-aged populations under 59 years old, while tracheal/bronchus/lung cancer**;** prostate cancer**;** colon and rectum cancer and gastric cancer demonstrated significantly heightened contributions to the cancer burden in elderly populations above 60 years old (**Figure 2B**). To further delineate the cancer burden within each age groups of the MAOs, we conducted stratified analysis of the top 10 cancers ranked by incidence rate in respective cohorts. Breast cancer remained the most common malignancy in the 40–59 years middle-aged groups (**Figures 2C**, **D**). With advancing age, tracheal/bronchus/lung cancer became the dominant cancer type in the 60–79 years strata (**Figures 2E, F**), while colorectal cancer ranked first in the ≥80 years group. (**Figure 2G**). Overall, tracheal/bronchus/lung; colorectal; and prostate cancers drove the majority of the cancer burden in adults aged ≥60 years. Given the prominent contribution of female-predominant malignancies in middle-aged populations, we further performed gender-stratified analysis (**Supplementary Figure S5**). In women aged ≥40 years, breast cancer was the most common malignancy across all age strata. Cervical; colorectal; and uterine cancers showed high incidence in middle-aged women, with breast; tracheal/bronchus/lung; and cervical cancers as the top three mortality contributors in this demographic (**Supplementary Figure S5A** left). In elderly women (≥60 years), the burdens of colorectal, tracheal/bronchus/lung; and stomach cancer increased markedly, and tracheal/bronchus/lung cancer concurrently emerged as the leading cause of cancer mortality (**Supplementary Figure S5A** right). In men, the predominant cancers in middle-aged population included colon and rectum**;** tracheal, bronchus and lung**;** gastric**;** prostate and liver cancers (**Supplementary Figure S5B** left). For elderly males, tracheal, bronchus, and lung cancer and prostate cancer had the two highest incidence rates(**Supplementary Figure S5B** right). Notably, within the male MAOs, tracheal/bronchus/lung cancer constituted the leading cause of cancer-related mortality. Thus, distinct oncological profiles exhibit marked heterogeneity across age strata and gender. The formulation of cancer prevention strategies necessitates systematic integration of age-specific stratification frameworks.

**Figure 2:**
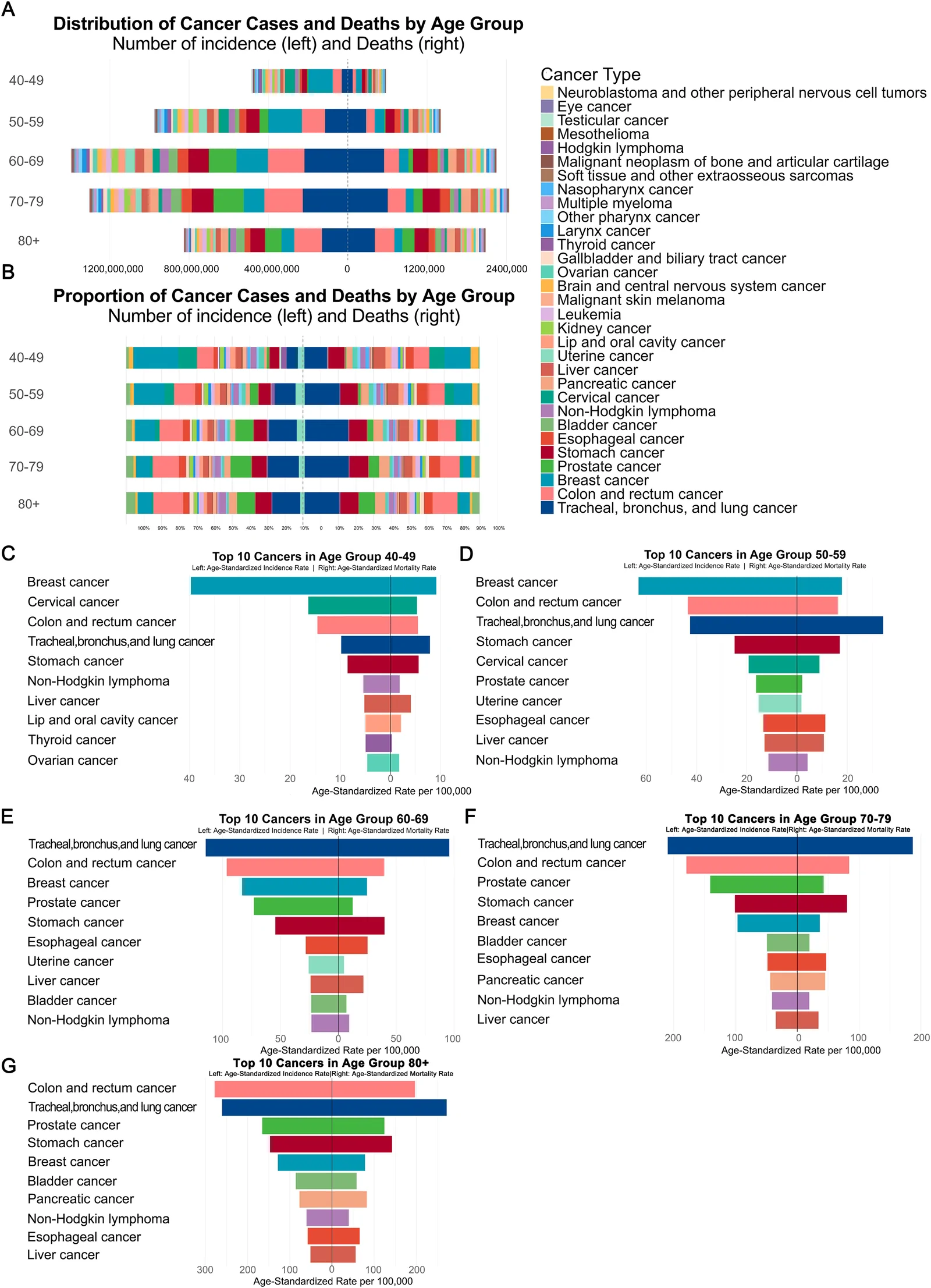
Age-stratified cancer burden profile. **(A)** Age-stratified presentation of case and death counts across cancers. **(B)** Age-stratified presentation of incidence proportions and mortality proportions across cancers. **(C–G)** The incidence and mortality of common cancers in age 40-49 **(C)**, 50-59 **(D)**, 60-69 **(E)**, 70-79 **(F)** and 80+ **(G)**.

### Differential cancer burden across SDI regions in MAOs

In 2021, the cancer burden among the MAOs also showed marked differences in various SDI regions. Overall, high SDI regions bore the heaviest cancer burden, and as the SDI index declined, the corresponding cancer burden also decreased, with low SDI regions having the lowest cancer burden (**Figure 3A**). Sex-specific analysis showed that female ASIR was higher than male ASIR across all five SDI quintiles in 2021. The female-to-male ASIR ratio was 1.028 in the high-SDI quintile, 1.153 in the high-middle-SDI quintile, 1.202 in the middle-SDI quintile, 1.443 in the low-middle-SDI quintile, and 1.489 in the low-SDI quintile. Thus, the relative female excess increased progressively with decreasing SDI, although the absolute ASIRs remained highest in the high-SDI quintile (**Supplementary Table S3**).The ranking of the top 10 cancers in MAOs varied in different SDI regions. Tracheal/bronchus/lung cancer**;** colon and rectum cancer and breast cancer consistently emerged as predominant contributors across almost all SDI regions. Colon and rectum cancer accounted for the highest proportion of incident cases in high-SDI regions, while tracheal, bronchus and lung cancer ranked first in both high-middle and middle-SDI regions. Breast cancer demonstrated the most substantial burden in low-middle and low-SDI regions (**Figure 3B**). A gradual decline in incident cases of the top 10 malignancies was observed with decreasing SDI. High-SDI regions exhibited the highest number of cases in tracheal, bronchus and lung cancer**;** colon and rectum cancer**;** breast cancer**;** prostate cancer**;** bladder cancer**;** non-Hodgkin lymphoma and pancreatic cancer, which generally decreased as the SDI declines. Inversely, cervical cancer presented the highest number of new cases in low-SDI regions and the lowest new cases in high-SDI regions (**Figure 3C**). When conducting a more granular comparison across SDI regions, the cancers contributing the most to the incidence burden in high-SDI regions were colon and rectum cancer**;** tracheal/bronchus/lung cancer**;** breast cancer and prostate cancer, with ASIRs being 145.30, 129.64, 122.72 and 122.16 per 100,000, respectively (**Figure 3D**; **Supplementary Table S2**). The main contributor to the mortality burden was tracheal/bronchus/lung cancer with an ASMR of 104.59 per 100,000 (**Supplementary Table S7**). As SDI decreased, the cancers with the highest ASIR gradually shifted from colon and rectum cancer and tracheal/bronchus/lung cancer in high-SDI regions to breast cancer and cervical cancer in low-SDI regions. In low-SDI regions, the main contributors to the cancer burden were breast**;** cervical and stomach cancer with ASIR of 30.65, 30.06 and 18.3 per 100,000 and ASMR of 19.73, 20.13 and 18.47 per 100,000, respectively (**Figure 3D**).

**Figure 3:**
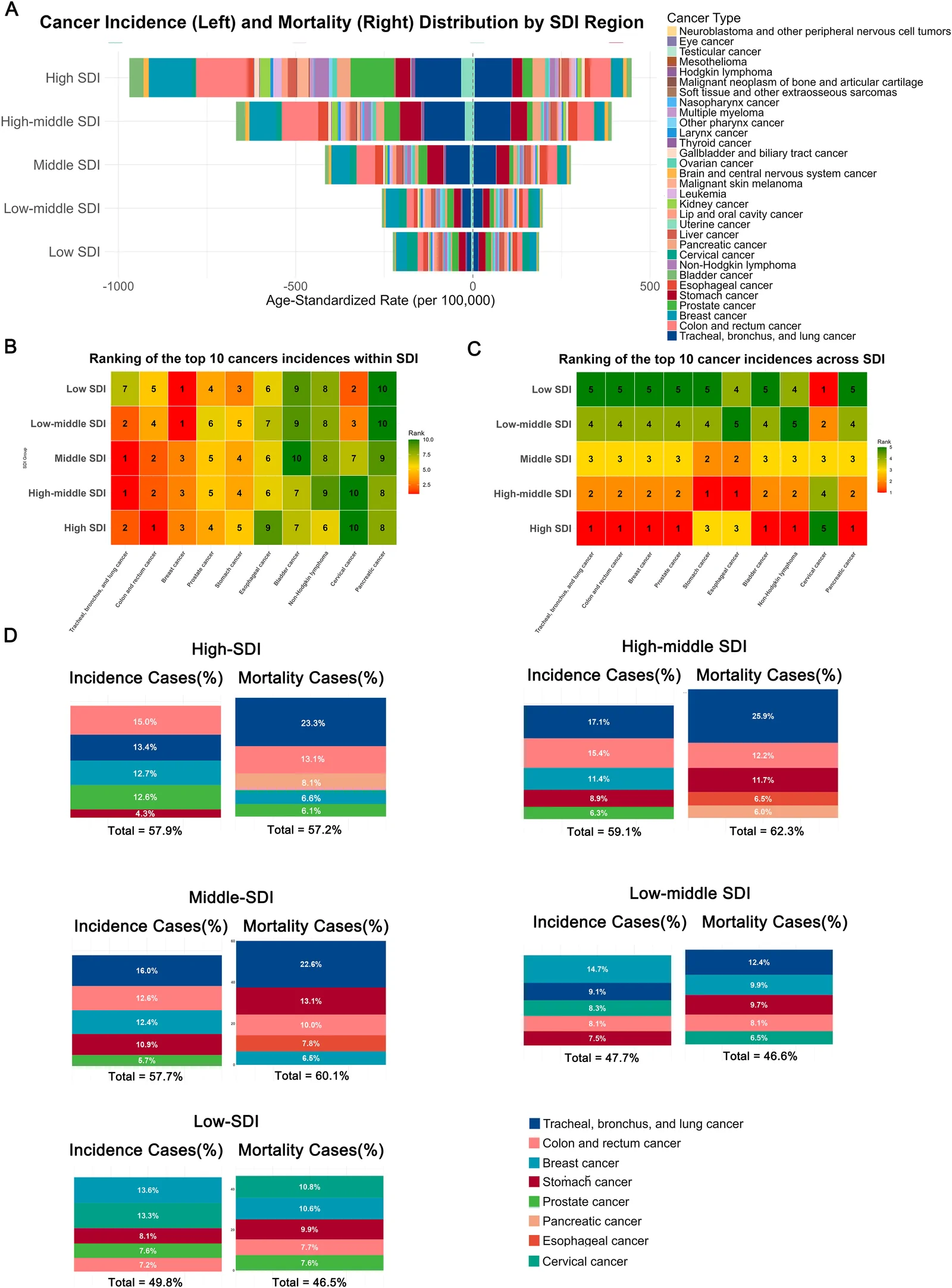
SDI-stratified cancer burden profile. **(A)**. SDI-stratified incidence and mortality of cancers. **(B)**. Rankings of the top 10 most common cancers within SDI. **(C)**. Rankings of the top 10 most common cancers across SDI. **(D)**. Proportion of the top five cancers in incidence and mortality cases across SDI regions.

### Differential cancer burden across 21 regions in MAOs

21 regions were divided from 5 SDI regions based on geographical differences and characteristics. The cancer burden of MAOs analyzed based on regions was generally similar as the results based on SDI regions. As **Figure 4A** shown, tracheal/bronchus/lung cancer and colon and rectum cancer were major challenges in High-income regions like High-income Asia Pacific, High-income North America and Western Europe. Breast cancer**;** cervical cancer and liver cancer had more impacts in low- and middle-income regions, including central sub-Saharan Africa, South Asia and Southeast Asia (**Figure 4A**). Regionally, the top 10 cancers largely mirrored the earlier findings, with tracheal/bronchus/lung cancer, colon and rectum cancer, breast cancer exhibiting high ASIR in almost all regions, while cervical cancer demonstrated high ASIR in low-SDI regions (**Figure 4B**). For the top 10 cancers by incidence among MAOs, significant regional disparities in cancer burden were observed. High-SDI regions, such as High-income Asia Pacific, exhibited a relatively higher cancer burden as ASIR. As the SDI level decreasing, the burden gradually decreased, with regions such as Western Sub-Saharan Africa and Eastern Sub-Saharan Africa disproportionately bearing the lightest burden (**Figure 4C**).

**Figure 4:**
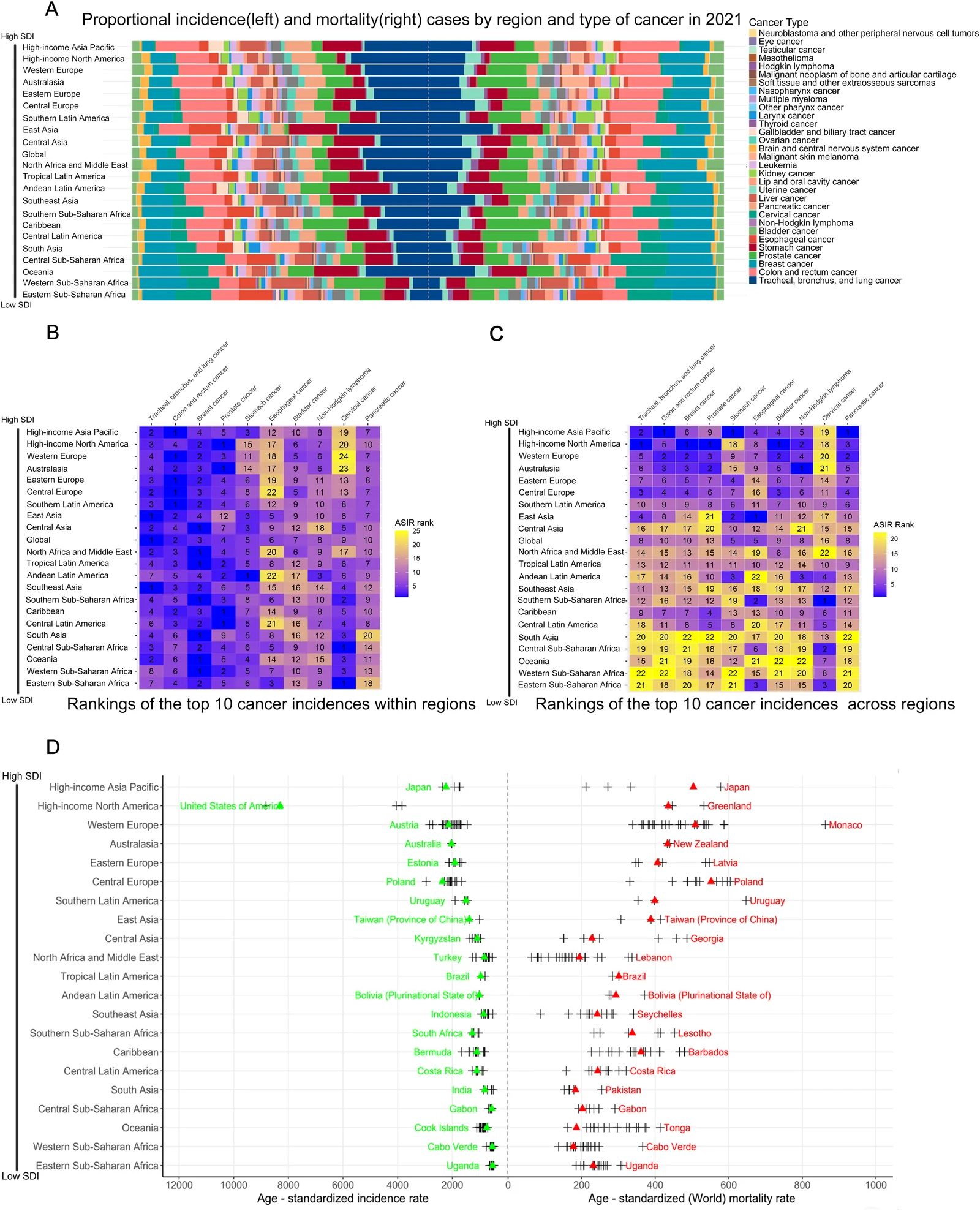
Region-stratified cancer burden profile. **(A)** Proportional contribution of cancers in incidence and mortality by region. **(B)** Rankings of the top 10 cancers within 21 regions. **(C)** Rankings of the top 10 cancers across 21 regions. **(D)** Distribution of Age-standardized incidence rate and age-standardized mortality rate of 204 countries.

Across the 21 GBD regions, female ASIR exceeded male ASIR in 19 regions, whereas male ASIR was higher in High-income North America and Australasia. The female-to-male ASIR ratio ranged from 0.938 in High-income North America to 1.614 in Oceania. At the global level, female ASIR was also higher than male ASIR, with a ratio of 1.139. The complete Sex-Specific estimates and their corresponding 95% UIs are presented in **Supplementary Table S4**. In many countries, the ASIR in MAOs did not change significantly with SDI, but the ASMR increased with rising SDI (**Figure 4D**).

The global map of ASIR and ASMR demonstrated the cancer burden of MAOs at the national level. As **Figure 5A** shown, higher ASIR and ASMR were concentrated in North America and Western Europe, while sub-Saharan Africa and South Asia faced lower ASIR. United States possessed the highest ASIR of 8,818.46 per 100,000 (95% UI, 7,178.11–10,971.89), while Monaco demonstrated the highest ASMR of 862.62 per 100,000 (95% UI, 698.57–1,030.53; **Supplementary Table S9**). Among MAOs, breast and prostate cancers were the most common in countries such as Germany, Australia and the United States, primarily located in High-income North America and High-income Asia Pacific. Tracheal, bronchus and lung cancer is the most frequently occurring tumor in China, Greenland, Turkey, Indonesia and so on, mainly in East Asia and High-income North America. Meanwhile, colon and rectum cancer had the highest ASIR in Northern Europe and North America (**Figure 5B** left). However, when analyzing mortality, we found that tracheal, bronchus and lung cancer was the leading cause of cancer-related death in 54% of countries globally within the MAOs (**Figure 5B** right).

**Figure 5:**
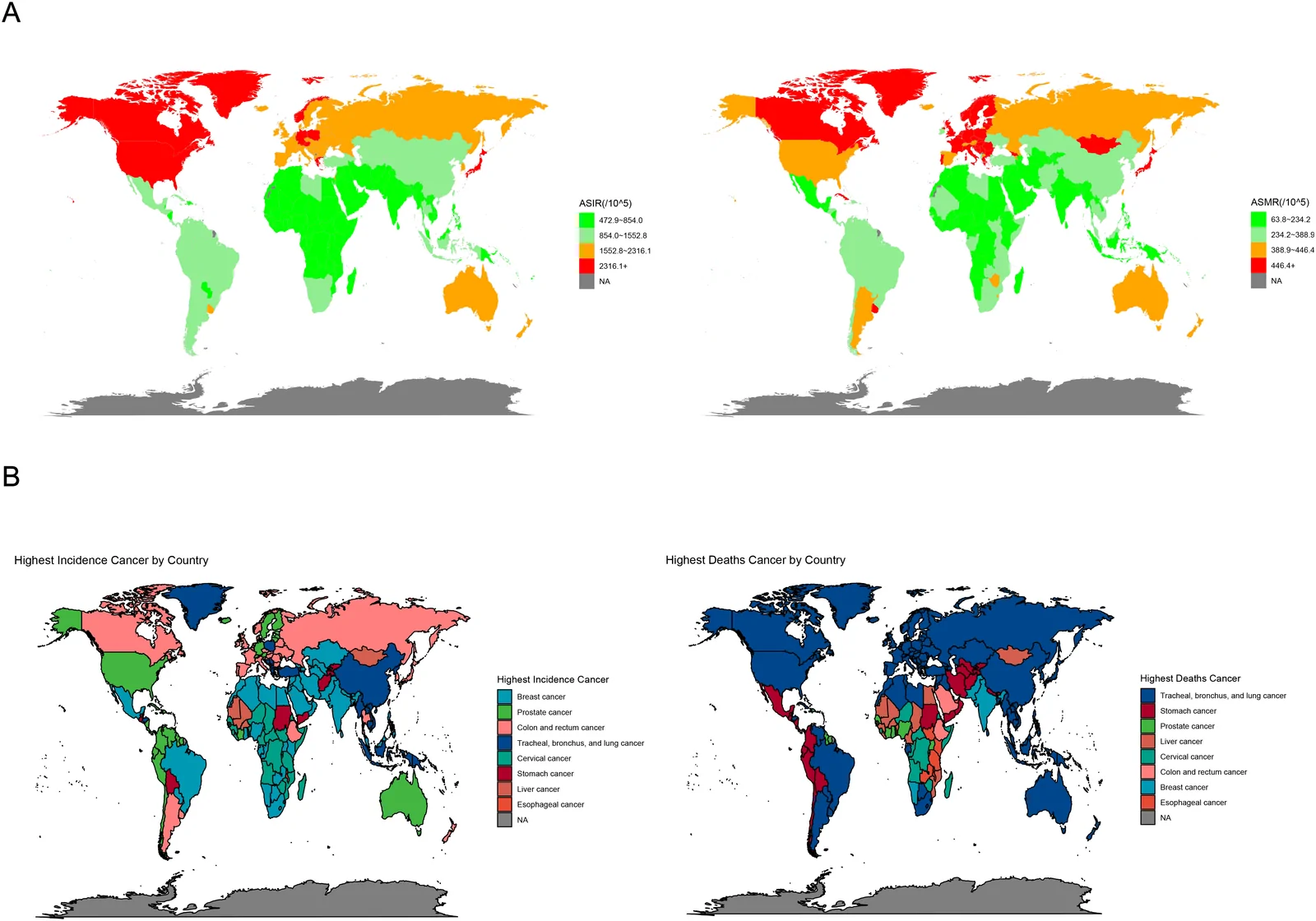
Country-stratified cancer burden profile. **(A)**. Cross-national comparison of cancer incidence and mortality. **(B)**. Leading cancers in incidence and mortality across nations.

### Attributable risk factors of cancers in MAOs

To inform cancer-prevention priorities, we analyzed the attributable risk factors for cancers based on GBD 2021 of MAOs globally. Collectively, the level 2 risk factor-attributable DALYs underwent significant changes over the decade from 2010 to 2021 and household air pollution from solid fuels dropped out of top ten, replaced by a diet low in milk. The burdens of ambient particulate matter pollution, high BMI, high fasting plasma glucose and diet low in milk increased, rising by 1.58%, 12.39%, 11.36% and 3.64%, respectively. In contrast, the attributions of most behavioral risk factors such as smoking, alcohol use, unsafe sex, diet high in red meat and diet low in whole grains decreased, dropping by 12.04%, 4.09%, 4.59%, 1.45% and 1.62% respectively (**Figure 6A**), **Figure 6B** presented the attributable proportion of the top 10 risk factors in the 10 most common cancers in 2021, measured by DALYs. Smoking contributed to over 60% of the attributable risk for tracheal, bronchus, and lung cancer. Colon and rectum cancer was primarily driven by dietary risks. Breast cancer was significantly associated with high BMI. Prstate, stomach, esophageal and bladder cancers mainly associated with smoking based on GBD database. Unsafe sex accounted for over 90% of the risk for cervical cancer and pancreatic cancer was associated with elevated fasting plasma glucose. Considering the feasibility of policy implementation and the availability of data, further PAF-based counterfactual analysis was conducted between six cancers and their corresponding risk factors. Based on GBD 2021 PAF estimates and a hypothetical 30% reduction in exposure relative to 2021 levels, the potential reductions in ASIR for six cancers among MAOs were estimated. For tracheal, bronchus, and lung cancer, ASIR could be reduced by approximately 16.9% by addressing smoking, 4.6% through control of ambient particulate matter pollution and 1.4% by reducing occupational exposure to asbestos (**Figure 6C**). For colon and rectum cancer, ASIR could be decreased by approximately 5.3% by increasing whole grains consumption, 4.7% by increasing milk intake, 4.4% by reducing a diet high in red meat (**Figure 6D**). For breast cancer, ASIR could be lowered by about 3.6% by reducing a diet high in red meat, 1.5% by managing high BMI, and 1.2% by controlling high fasting plasma glucose (**Figure 6E**). For stomach cancer, ASIR could be reduced by about 3.2% by controlling smoking (**Figure 6F**). For esophageal cancer, ASIR could be reduced by about 9.8% by reducing smoking and 5.2% by controlling high alcohol use (**Figure 6G**). For pancreatic cancer, ASIR could be lowered by approximately 6.1% by controlling high fasting plasma glucose, 5.2% by reducing smoking and 0.6% by managing high BMI (**Figure 6H**). To further clarify the reasons behind the differences in cancer profiles between high- and low-SDI regions, we analyzed attributable risk factors based on the SDI. Overall, from 2010 to 2021, behavioral risks contributed less to the cancer burden in both low- and high-SDI regions, while metabolic risks exhibited an upward trend, particularly in high-SDI areas. In high-SDI regions, the largest declines in contributions to the cancer burden were observed for ambient particulate matter pollution (-27.42%), smoking (-17.82%), unsafe sex (-14.82%), and occupational exposure to asbestos (-12.65%). Despite these reductions, smoking remained the leading risk factor for cancer burden (**Supplementary Figure S6A**). Conversely, high BMI (+5.79%) and high fasting plasma glucose (+11.12%) showed marked increases, ranking as the second and third most impactful attributable risk factors in 2021. In contrast, low-SDI regions experienced minimal shifts in attributable risk factors during the same period. Unsafe sex, smoking, alcohol use, and household air pollution from solid fuels persisted as the top four risk factors, albeit with slight declines over the 11-year span (**Supplementary Figure S6B**). The only notable increasing risk factors for cancer DALYs were linked to high BMI, high fasting plasma glucose, and diet high in red meat.

**Figure 6:**
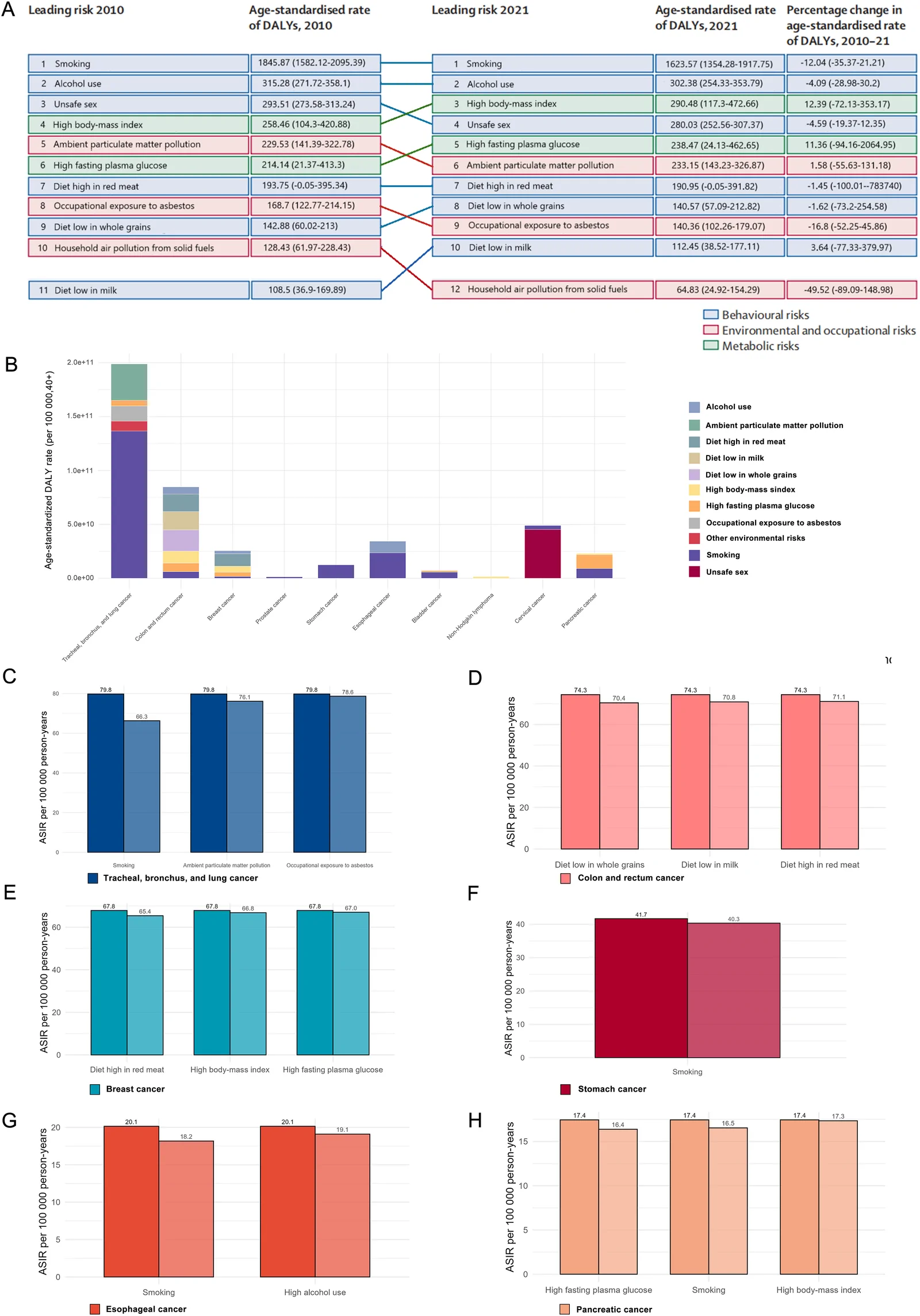
Epidemiological profile of cancer risk factors. **(A)** Changes of age-standardized DALY rate for risk-attributable cancer burden from 2010 to 2021. **(B)** Proportional of risk factors attributed to common cancers. **(C–H)** Causal Chain Analysis of Risk Factors Associated with Common Cancers, including tracheal, bronchus, and lung cancer **(C)**, colon and rectum cancer **(D)**, breast cancer (E), stomach cancer (F), esophageal cancer (G), and pancreatic cancer (H).

## Discussion

This study utilized the GBD database to comprehensively analyze the cancer burden among MAOs (40 years and older) worldwide. A detailed evaluation was conducted on disparities in cancer incidence and mortality across different age groups, SDI regions and genders. The findings revealed that the cancer burden escalated with age, but distinct cancer profiles emerged across different regions. High-SDI regions, particularly High-income North America, exhibited a significant upward trend in cancer incidence, likely attributable to lifestyle factors, environmental exposures, and healthcare accessibility. Globally, while cancer incidence and prevalence increased from 1990 to 2021, mortality and DALYs declined markedly, reflecting advancements in treatment strategies and improved patient prognoses. Furthermore, this study highlighted the dual impact of behavioral and metabolic risk factors on cancer burden, providing critical insights for refining precision-based cancer prevention and control strategies. Incident cancer cases among adults aged ≥40 years were projected to increase by 39.6% by 2035, driven predominantly by population growth (74.8%), followed by population aging (17.1%) and changes in age-specific incidence rates (8.1%). These findings highlight the need to expand cancer-care capacity, particularly in rapidly growing low-SDI settings; however, our analysis cannot directly quantify the additional workforce, equipment, or hospital beds required (16). Nevertheless, the ARIMA projections should be interpreted cautiously, as they extrapolate the temporal patterns observed during 1990–2021 and do not explicitly account for future changes in cancer prevention, screening, diagnosis, treatment, risk-factor exposure, health policy, or disruptive events (17).

For middle-aged women (40–59 years), breast cancer and cervical cancer exhibited the highest incidence rates, with pronounced regional disparities. Both cancers demonstrated substantially higher burdens in low-SDI regions, decreasing progressively with higher SDI levels, which is consistent with prior reports. Multiple studies have confirmed that breast and cervical cancers remained the most prevalent malignancies in women (9, 12, 18, 19). According to the latest International Agency for Research on Cancer (IARC) data, breast cancer retained its position as the leading cancer among women globally in 2022, with ASIRs of 54.1 per 100,000 in transitioning countries and 30.8 per 100,000 in transitioned countries (20). Elevated ASIR in high-SDI regions correlate with advanced diagnostic modalities, such as mammography and breast magnetic resonance imaging (MRI). Our age-stratified data showed that breast cancer remained the leading cause of cancer death in women aged 40–49 years. These findings carry implications for breast cancer screening policy. Currently, most international guidelines recommend routine mammography for women aged 50–69 years as standard practice. Whether to extend population screening to the 40–49 age group remains debated due to concerns over higher false-positive rates and overdiagnosis. Nonetheless, a growing number of High-income countries have piloted screening starting at 40–45 years, with evidence supporting mortality benefits in high-risk subgroups. For high-SDI regions with adequate healthcare capacity, risk-stratified targeted screening for women aged 40–49 years may be feasible. For low- and middle-SDI regions, priority should be given to universal screening coverage for the 50–69 age group, complemented by breast health education for younger middle-aged women. Cervical cancer, strongly associated with human papillomavirus (HPV) infection, disproportionately affected low-SDI regions due to insufficient public health resources, low vaccination coverage, and limited early screening awareness (21–23). Additionally, unsafe sexual practices constituted a primary attributable risk factor in these regions. Consequently, enhancing vaccination programs, targeted cancer screening, early diagnosis, standardized treatment, and public health education are pivotal for reducing the cancer burden in low-SDI areas. Notably, cervical cancer burden declined significantly among older women (≥70 years), suggesting that its higher incidence in middle-aged women correlates with sexual activity levels. Thus, breast and cervical cancers prevention should be prioritized for middle-aged women globally, while colorectal and tracheal/bronchus/lung cancers warrant greater attention in older women.

Among male MAOs, colorectal cancer; tracheal/bronchus/lung cancer; gastric cancer; prostate cancer and esophageal cancer represented key prevention targets. Prostate cancer incidence raised sharply in men aged≥60 years, particularly in Australasia, South America and Asia, consistence with previous studies (24, 25). Notably, the elevated prostate cancer incidence in high-SDI countries can also be attributed to the widespread implementation of opportunistic prostate-specific antigen (PSA)-based screening, which increases detection of early-stage asymptomatic cases and elevates the overall reported incidence. Beyond age, ethnicity, family history and genetic predisposition (26), modifiable risk factors such as smoking, obesity, sedentary behavior, red meat consumption, chronic prostatitis and HPV infection contribute to prostate cancer development (25, 27–29). Mitigating these lifestyle factors is critical for prevention. In middle-aged men, the incidence and mortality of liver cancer remained elevated. Recent stabilization in global liver cancer rates stems from reduced aflatoxin exposure in agriculture, widespread hepatitis B vaccination, and antiviral therapies (30, 31). However, rising non-viral hepatocellular carcinoma linked to metabolic dysfunction-associated steatotic liver disease (MASLD) may supplant viral hepatitis as the primary risk factor (32), necessitating lifestyle-focused interventions.

Tracheal, bronchus, and lung cancer persists as a global priority for cancer control. Our data indicated that tracheal, bronchus, and lung cancer ranked the first in incidence in China, Greenland, Turkey, Indonesia and so on. At the national level, tracheal, bronchus, and lung cancer dominated mortality rates in most middle- and high-SDI countries. Smoking remained the leading risk factor, followed by air pollution and occupational hazards. A GBD-based study underscored smoking, secondhand smoke, elevated fasting plasma glucose, and occupational exposures as critical targets for racheal, bronchus, and lung cancer prevention (33). Similarly, our study demonstrated that smoking contributed to over 60% of the attributable risk for tracheal, bronchus, and lung cancer. The ASIR could decrease 16.9% if smoking exposure reduce 30%. Hence, active smoking cessation and prohibition are the key to reduce tracheal, bronchus, and lung cancer.

Finally, to identify global priorities, we analyzed attributable risk factors. Analysis of cancer risk factors in MAOs reveals that from 2010 to 2021, the contribution of risk factors was characterized by “rising metabolic risks and differentiated behavioral risks” with significant regional disparities: in high-SDI regions, metabolic risks such as high BMI (5.79% increase) and high fasting plasma glucose (11.12% increase) rose notably, becoming core risks second only to smoking; in low-SDI regions, behavioral risks including unsafe sex, smoking, and household air pollution from solid fuels remained dominant, with only a slight rise in metabolic risks, reflecting health challenges at different development stages. These increases reflect modeled risk-attributable burden and may partly be influenced by GBD 2021 methodological updates, but they nevertheless support prioritizing obesity prevention and diabetes management, particularly in high-SDI settings (34). Robust evidence links these factors to multiple cancers (35–38). In terms of cancer-specific associations, smoking contributed over 60% to tracheal, bronchus, and lung cancer, and a 30% reduction in smoking exposure could lower the ASIR of this cancer by 16.9%; colorectal cancer was strongly influenced by diet, with significant intervention potential from increasing whole grain intake (5.3% ASIR reduction) and decreasing red meat consumption (4.4% ASIR reduction); over 90% of cervical cancer risk stemmed from unsafe sex, while breast cancer was closely linked to high BMI and pancreatic cancer to high fasting plasma glucose. These PAF-based estimates should be interpreted as standardized population-level counterfactual potentials rather than immediately attainable policy effects or individual-level risk reductions, because achieving a 30% exposure reduction may require sustained, context-specific interventions over years or decades, while the underlying GBD Comparative Risk Assessment estimates depend on causal assumptions and may be affected by residual confounding and exposure-measurement error (34, 39). Base on the analysis, precision regional strategies are required for prevention and control. High-SDI regions should manage metabolic risks through nutrition education and exercise promotion while strengthening tobacco and alcohol control. Low-SDI regions should prioritize HPV vaccination, cervical cancer screening, screening for cervical, breast, and colorectal cancers, clean energy popularization, and enhanced tobacco/alcohol control. Globally, tobacco control should be the key focus for tracheal, bronchus, and lung cancer as for the leading cancer killer in 54% of countries, with high-SDI regions also addressing PM2.5 control and low-SDI regions focusing on occupational carcinogen protection, to reduce the cancer burden in MAOs via differentiated strategies.

We also noticed the inconsistent gender pattern of overall cancer incidence among people aged over 40 years compared with traditional epidemiological consensus and recent IARC statistics. Traditionally and in most High-income regional surveys, males tend to suffer from higher total cancer incidence than females in middle-aged and elderly populations. The opposite or narrowed gender gap observed in our GBD-based global analysis can be reasonably explained. On the one hand, the high prevalence of breast cancer and cervical cancer substantially increases the overall cancer burden in females aged 40 years and older. Female ASIR exceeded male ASIR across all five SDI quintiles and in 19 of the 21 GBD regions, whereas male ASIR remained higher in High-income North America and Australasia. This pattern may reflect differences in cancer-site composition and regional distributions rather than universally greater female susceptibility, while variation in data availability and diagnostic ascertainment across cancer sites and settings may also influence pooled GBD estimates, particularly in data-sparse regions (40, 41).

To our knowledge, this is the first study focusing on the global cancer burden among MAOs. Nevertheless, several limitations should be acknowledged. First, we defined MAOs as individuals aged ≥40 years based on the conventional AYA definition of 15–39 years, with 40 years representing the age immediately above the established AYA range. Although our analyses demonstrated distinct cancer profiles in both the 40–49 and 50–59 year age groups, adults aged 40–49 years are also included in studies of early-onset cancer. Therefore, comparisons with studies using different age definitions should be interpreted with caution (42, 43). Second, country-level estimates for microstates, such as Monaco, should also be interpreted cautiously because sparse data may result in wide uncertainty intervals and unstable point estimates. However, these limitations are unlikely to materially affect regional or SDI-stratified estimates, which were derived independently at their respective aggregation levels (6).

## Conclusion

This study delineates the global burden and evolving trends of cancer among MAOs, clarifying gender-, age-, and region-specific distributions and SDI-associated risk factors. Breast, tracheal, bronchus and lung, colorectal, and cervical cancers remain paramount targets for prevention. Tailoring interventions to demographic and regional disparities will enhance resource allocation and strengthen cancer control strategies worldwide.

## Data Availability

The data used in this study were obtained from the Global Burden of Disease Study 2021 (GBD 2021). The datasets are publicly available via the GHDx (Global Health Data Exchange) repository at the following direct link: https://vizhub.healthdata.org/gbd-results/No specific accession number is assigned for GBD public aggregate data. All data were extracted from publicly accessible summary results provided by the Institute for Health Metrics and Evaluation (IHME).

## Glossary

MAOs: Middle-aged and older adults
GBD: Global Burden of Diseases
DALYs: Disability-Adjusted Life Years
ARIMA: autoregressive integrated moving average
SDI: Socio-demographic Index
ASIR: Age-standardized incidence rate
ASMR: Age-standardized mortality rate
SDI: Socio-demographic Index
WHO: World Health Organization
IARC: International Agency for Research on Cancer
AYAs: adolescents and young adults
ICD-10: International Classification of Diseases, 10th Revision
PM2.5: particulate matter with an aerodynamic diameter of ≤2.5 μm
TMRELs: theoretical minimum risk exposure levels
PAFs: Population attributable fractions
YLLs: years of life lost
YLDs: years lived with disability
ASRs: Age-standardized rates
ASPR: Age-standardized prevalence rate
ASDR: Age-standardized DALYs rate
GBD-WSPS: GBD World Standard Population Structure
PBCRs: Population-based cancer registries
CODEm: Cause of Death Ensemble modeling
HAQ: Healthcare Access and Quality
UIs: uncertainty intervals
EDM: Ensemble Distribution Modeling
APCs: annual percentage changes
AAPCs: average annual percentage changes
CIs: confidence intervals
ACF: autocorrelation function
PACF: partial autocorrelation function
AIC: Akaike Information Criterion
BIC: Bayesian information criterion
RMSE: root-mean-square error
AICc: corrected Akaike information criterion
MRI: magnetic resonance imaging
HPV: human papillomavirus
PSA: prostate-specific antigen
MASLD: metabolic dysfunction-associated steatotic liver disease.
