# Supplementary material for: Global, regional and national cancer burden and trends of middle-aged and older populations: a GBD 2021-based analysis, prediction and prevention
Source: Front Oncol. 2026 Oct 7;16:1859604. doi: 10.3389/fonc.2026.1859604 (PMC13683138; doi:10.3389/fonc.2026.1859604)
Supplement: Supplementary file 1 [file DataSheet1.pdf]

Figure S1

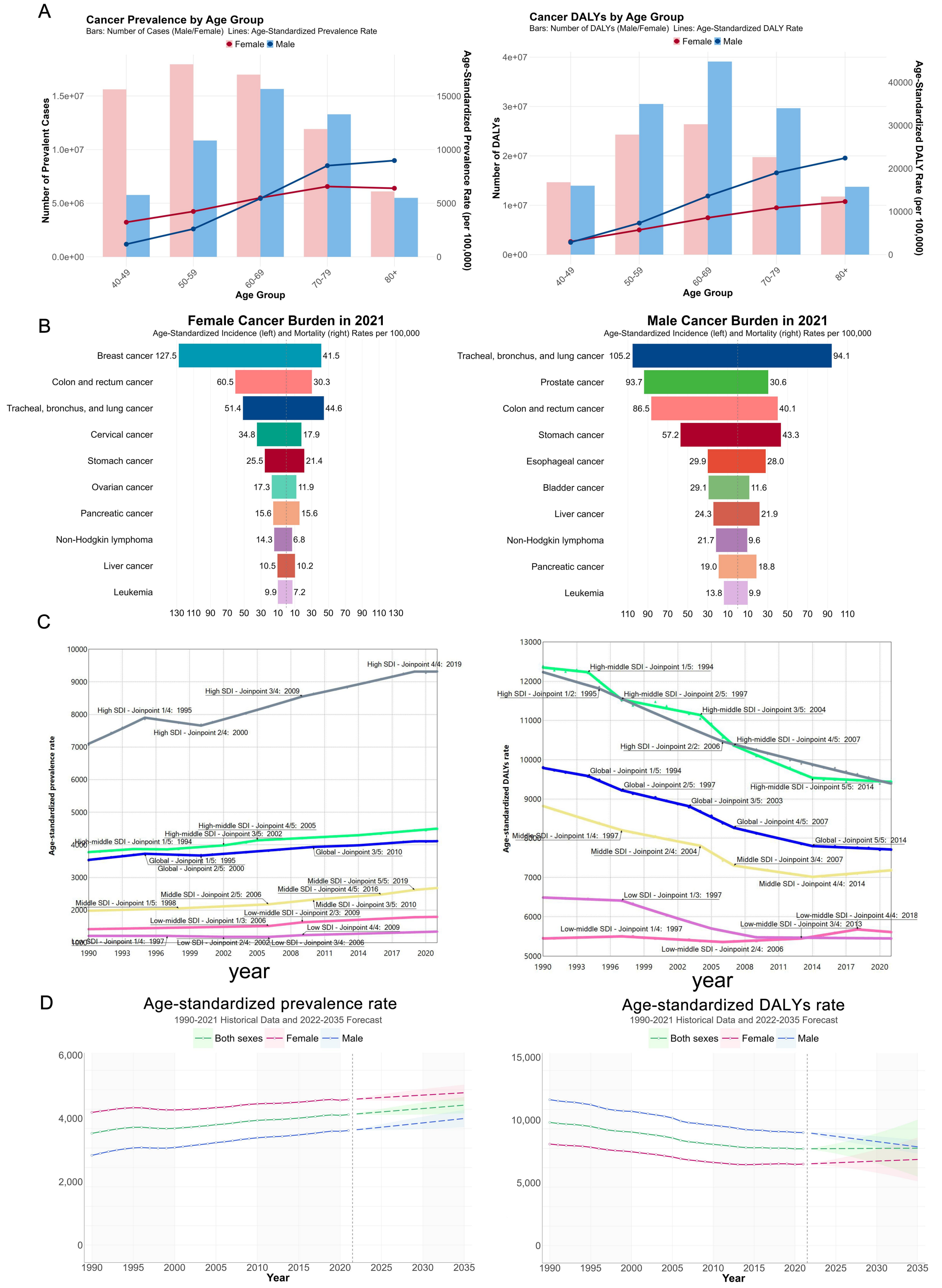

FigureS1 Cancer Burden of MAOs. A. Age- stratified cancer prevalent (left) and DALYs (right) cases and age-standardized prevalent rates (left) and age-standardized DALY rates (right) by sex. B. The incidence and mortality of

female (left) and male (right) of common cancers in 2021. C. Join point regression analysis of prevalence (left) and DALYs (right) by SDI. D. Sex-specific ARIMA projections of the global age-standardized prevalence rate (left) and age-standardized disability-adjusted life-year rate (right) from 2022 to 2035.

Figure S2

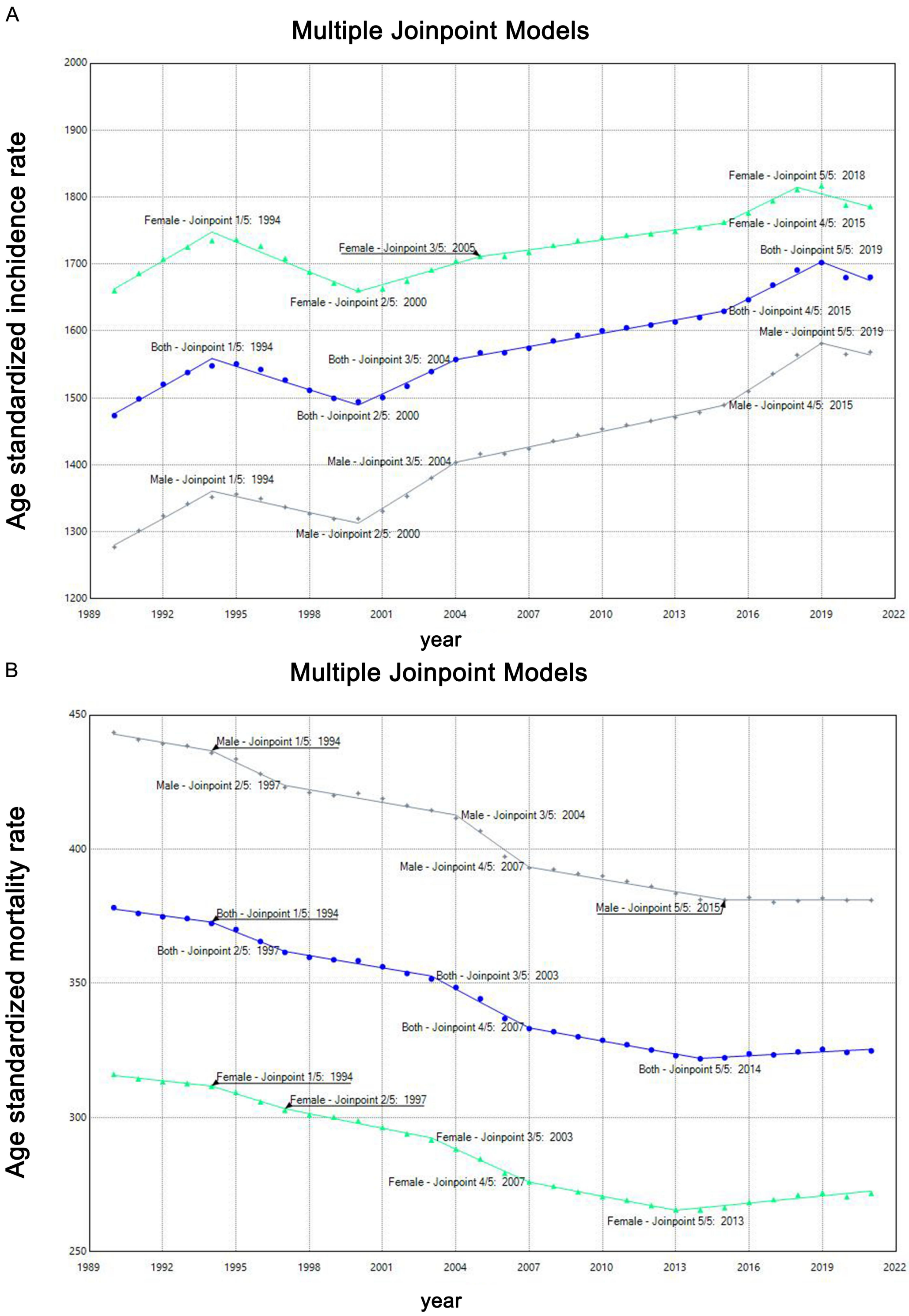

Figure S2. Sex-specific Joinpoint regression analysis of global cancer incidence and mortality among MAOs. A. Joinpoint regression analysis of the global age-standardized incidence rate by sex from 1990 to 2021. B.

Joinpoint regression analysis of the global age-standardized mortality rate by sex from 1990 to 2021. The labeled years indicate the joinpoints identified for males, females, and both sexes.

Figure S3

A

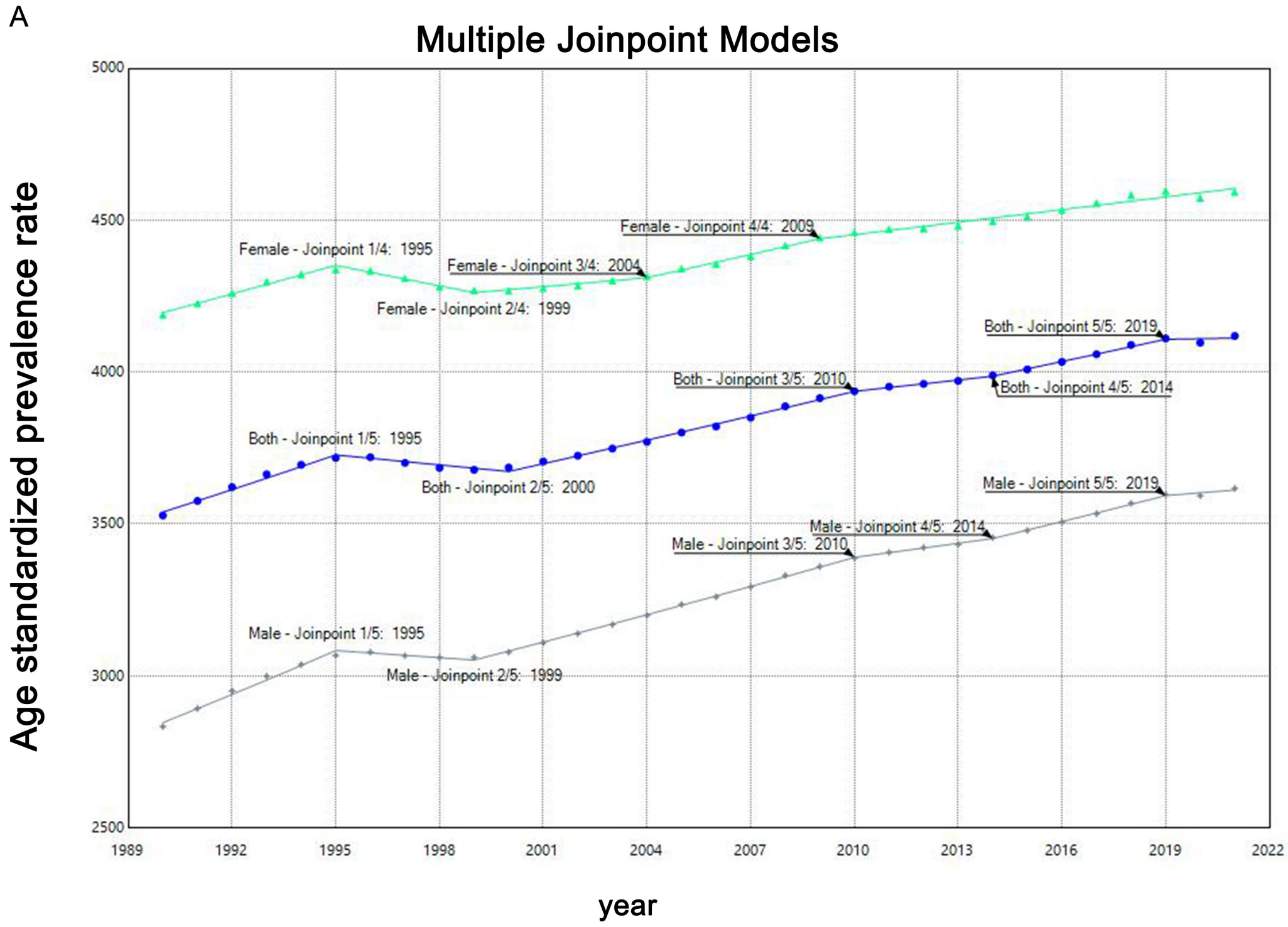
Multiple Joinpoint Models
Age standardized prevalence rate
year
Female - Joinpoint 1/4: 1995
Female - Joinpoint 2/4: 1999
Female - Joinpoint 3/4: 2004
Female - Joinpoint 4/4: 2009
Both - Joinpoint 1/5: 1995
Both - Joinpoint 2/5: 2000
Both - Joinpoint 3/5: 2010
Both - Joinpoint 4/5: 2014
Both - Joinpoint 5/5: 2019
Male - Joinpoint 1/5: 1995
Male - Joinpoint 2/5: 1999
Male - Joinpoint 3/5: 2010
Male - Joinpoint 4/5: 2014
Male - Joinpoint 5/5: 2019

B

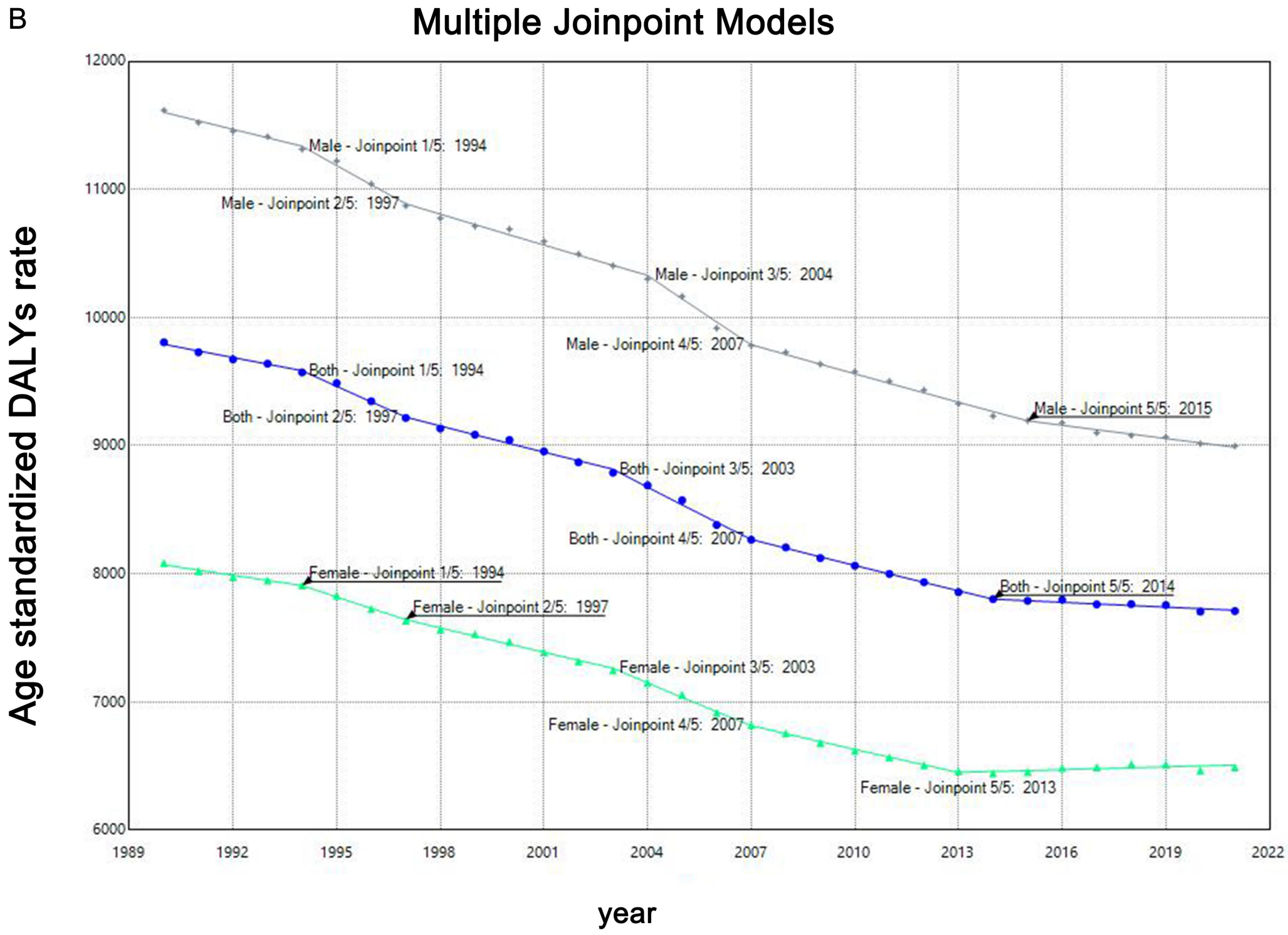
Multiple Joinpoint Models
Age standardized DALYs rate
year
Male - Joinpoint 1/5: 1994
Male - Joinpoint 2/5: 1997
Male - Joinpoint 3/5: 2004
Male - Joinpoint 4/5: 2007
Male - Joinpoint 5/5: 2015
Both - Joinpoint 1/5: 1994
Both - Joinpoint 2/5: 1997
Both - Joinpoint 3/5: 2003
Both - Joinpoint 4/5: 2007
Both - Joinpoint 5/5: 2014
Female - Joinpoint 1/5: 1994
Female - Joinpoint 2/5: 1997
Female - Joinpoint 3/5: 2003
Female - Joinpoint 4/5: 2007
Female - Joinpoint 5/5: 2013

Figure S3. Sex-specific Joinpoint regression analysis of global cancer prevalence and DALY burden among MAOs. A. Joinpoint regression analysis of the global age-standardized prevalence rate by sex from 1990 to 2021. B. Joinpoint regression analysis of the global age-standardized DALY rate by sex from 1990 to 2021. The labeled years indicate the joinpoints identified for males, females, and both sexes.

Figure S4

A

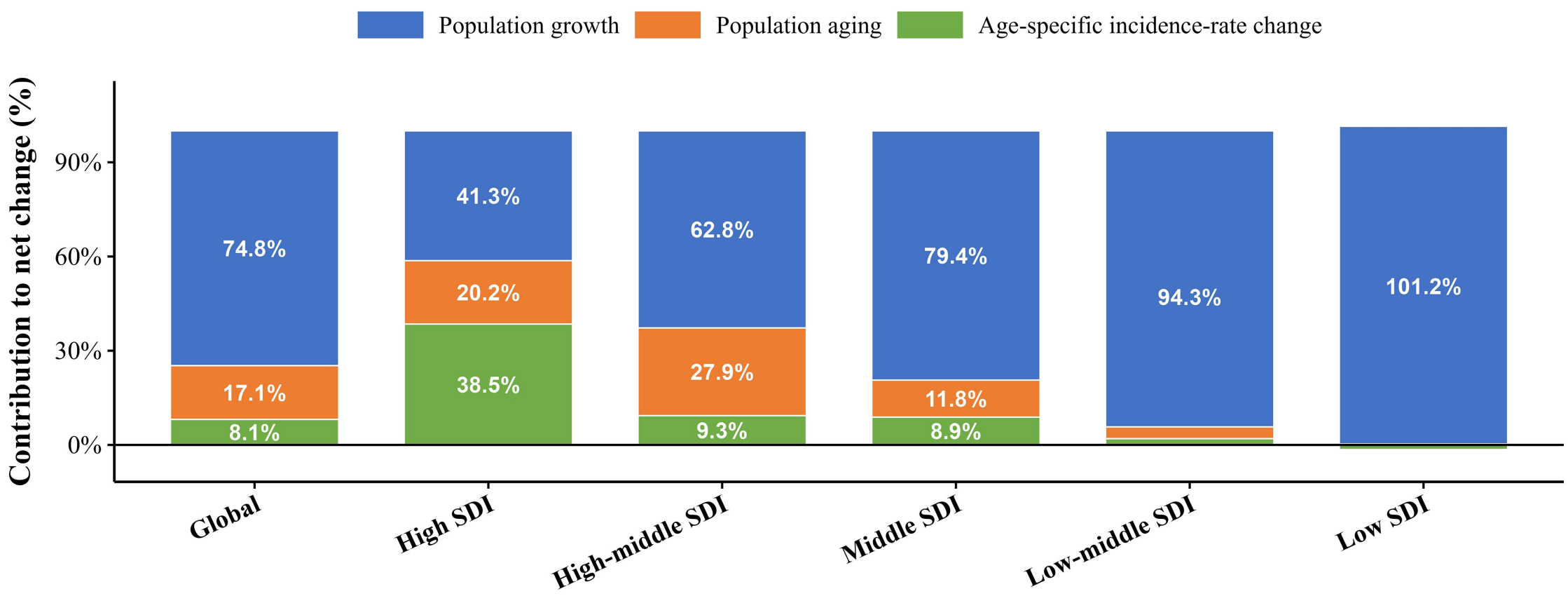

B

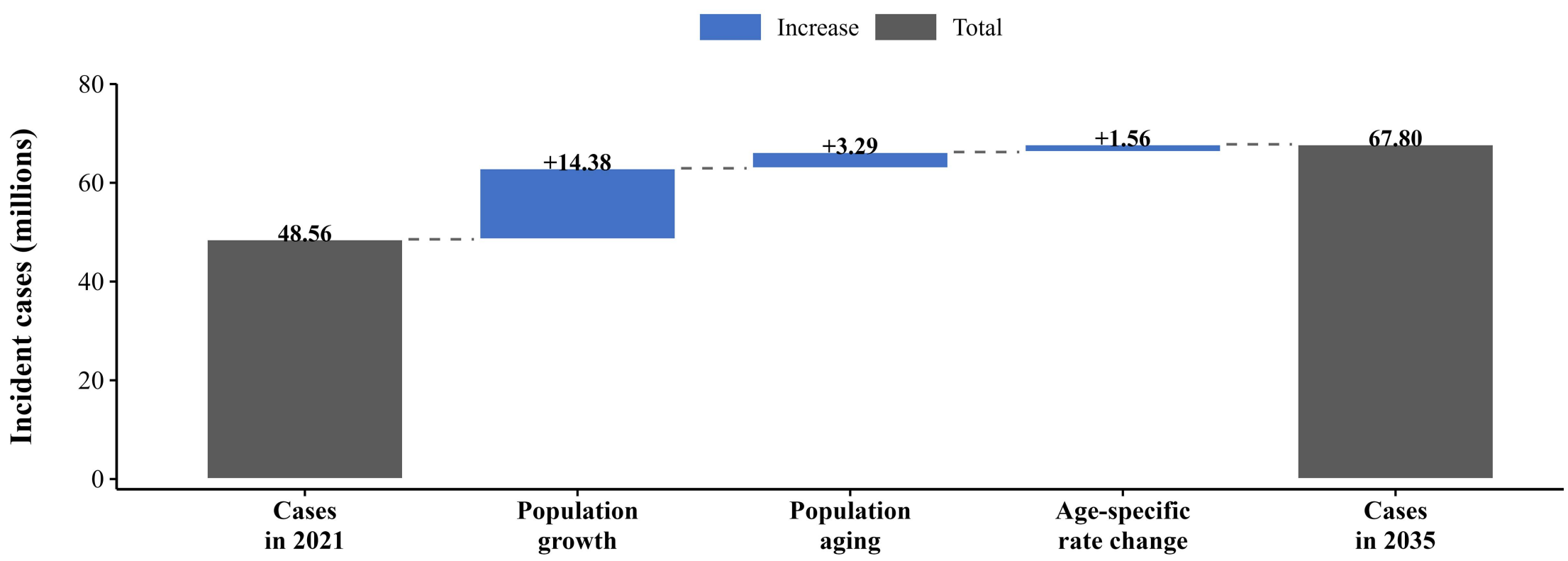

Figure S4 Decomposition of Projected Changes in Incident Cancer Cases. A. Relative contributions of population growth, population aging, and changes in age-specific incidence rates to the projected net change in incident cancer cases globally and across SDI quintiles. B. Absolute contributions of

population growth, population aging, and changes in age-specific incidence rates to the projected global increase in incident cancer cases from 2021 to 2035.

Figure S5

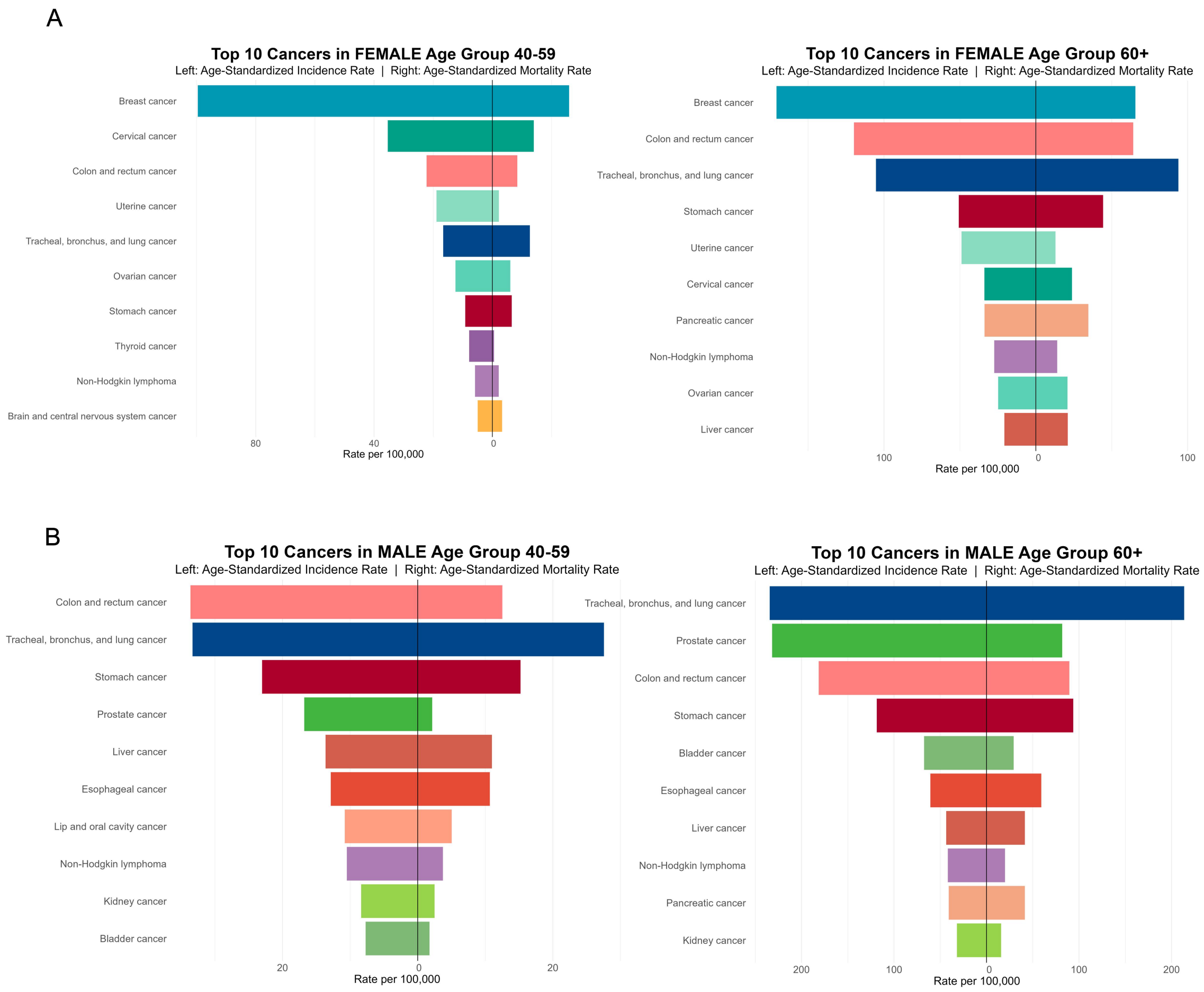

Figure S5 Sex- and age-Stratified Cancer Burden. A. Females' age-standardized incidence rate and age-standardized motality rate of the common cancers in age 40-59 (left) and 60+ (right). B. Males'

age-standardized incidence rate and age-standardized motality rate of the common cancers in age 40-59 (left) and 60+ (right).

Figure S6

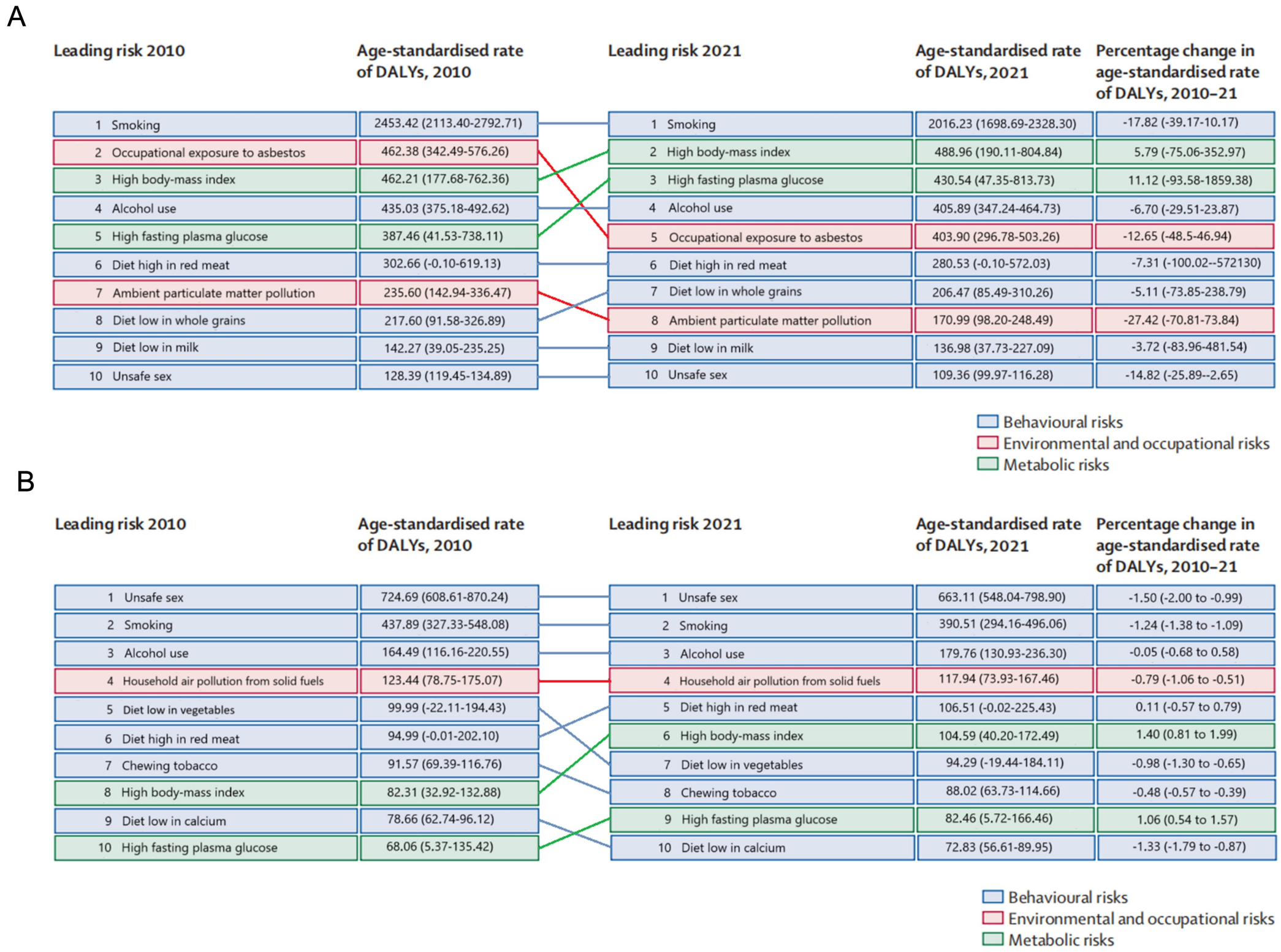

FigureS6 Comparison of Attributable Risk Factors between 2010 and 2021 by SDI. Changes of age-standardized DALY rate for risk-attributable cancer burden in high-SDI (A) and low-SDI (B) regions from 2010 to 2021.
